# Blockade of FGFR1 Trafficking to the Cell Surface Results in the Partial Mistargeting of the Receptor to Peroxisomes

**DOI:** 10.1096/fj.202600529RR

**Published:** 2026-07-01

**Authors:** Paulina Działek, Aleksandra Chorążewska, Martyna Biaduń, Jian Qiu, Natalia Porębska, Łukasz Opaliński

**Affiliations:** ^1^ Department of Medical Biotechnology, Faculty of Biotechnology University of Wroclaw Wroclaw Poland; ^2^ Department of Protein Engineering, Faculty of Biotechnology University of Wroclaw Wroclaw Poland; ^3^ Institute of Molecular Precision Medicine Xiangya Hospital, Central South University Changsha China

**Keywords:** DHRS2, ER, FGFR1, glycosylation, peroxisomes, protein transport

## Abstract

Fibroblast growth factor receptor 1 (FGFR1) is a cell surface receptor tyrosine kinase implicated in cellular signaling and homeostasis. Several reports indicate that N‐glycosylation of FGFR1 is critical for the FGFR1 trafficking to the cell surface, as glycosylation‐deficient mutant of FGFR1 (FGFR1.GF) is trapped inside the cell, in the endoplasmic reticulum (ER), and in the nuclear envelope. Our recent mass spectrometry analyses revealed dehydrogenase/reductase 2 (DHRS2) as a putative binding partner of the intracellular FGFR1.GF. Here, we identified a peroxisomal targeting signal 1 (PTS1) at the C‐terminus of DHRS2 and demonstrated that DHRS2 is dually targeted to peroxisomes and mitochondria. Furthermore, we determined that knockdown of DHRS2 results in increased number of peroxisomes, implicating the role of DHRS2 in peroxisome biogenesis. Using proximity ligation assay (PLA), we confirmed the interaction between FGFR1.GF and DHRS2 and demonstrated that FGFR1.GF/DHRS2 complexes are predominantly detected in peroxisomes. In agreement, we detected a small fraction of FGFR1.GF in peroxisomes. Taken together our data indicate that accumulation of FGFR1.GF in the ER may result in FGFR1.GF targeting to peroxisomes. Furthermore, we reveal interconnection between FGFR1 and DHRS2 and their role in peroxisome biogenesis.

## Graphical abstract

Fibroblast growth factor receptor 1 (FGFR1) is co‐translationally integrated into endoplasmic reticulum (ER) membranes and undergoes N‐glycosylation at several positions. This process is essential for FGFR1 progression through the secretory pathway. Impaired N‐glycosylation of FGFR1 (FGFR1.GF) leads to the intracellular accumulation of FGFR1 predominantly in the ER and in the nuclear envelope. Occasionally, vesicles that transport peroxisomal membrane proteins from the ER to peroxisomes include FGFR1.GF, which is highly abundant in the ER, delivering FGFR1.GF to peroxisomes.

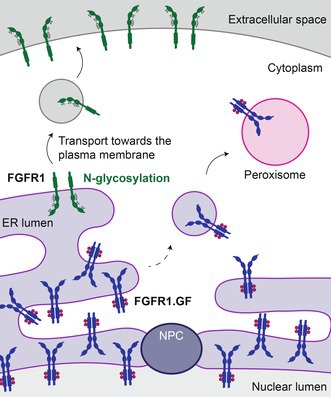

**Abbreviations**
BLI: bio‐layer interferometry
DHRS2: dehydrogenase/reductase SDR family member 2
ER: endoplasmic reticulum
FBS: fetal bovine serum
FGFR1: fibroblast growth factor receptor 1
FGFR1‐Fc: glycosylated extracellular domain of FGFR1 fused to the Fc antibody fragment
FGFR1.GF: N‐glycosylation‐deficient FGFR1
FGFR1‐KD: His‐tagged kinase domain of FGFR1
FGFRs: fibroblast growth factor receptors
FGFs: fibroblast growth factor factors
mGFP: methionine deficient green florescent protein
MS: mass spectrometry
MTS: mitochondrial targeting sequence
NPC: nuclear pore complex
PBS: phosphate‐buffered saline
PEX11β: peroxisomal biogenesis factor 11 beta
PEX14: peroxisomal membrane protein PEX14
PFA: paraformaldehyde
PLA: proximity ligation assay
PTS1: peroxisomal targeting signal
RT: room temperature
RTKs: receptor tyrosine kinases
SBP: streptavidin binding peptide
SD: standard deviation

## 1 Background

Fibroblast growth factor receptor 1 (FGFR1) together with fibroblast growth factors (FGFs) forms a signal transduction platform regulating fundamental cellular processes like apoptosis, differentiation, division, and motility [1, 2]. FGFR1 signaling is critical for the development of the human body [1]. Its dysregulation is observed in several disorders including lung, breast, head and neck, and urothelial cancers [1, 3, 4, 5, 6, 7, 8, 9]. FGFR1, as a member of the receptor tyrosine kinase (RTK) family, consists of an N‐terminal extracellular region including three immunoglobulin‐like domains D1–D3 enriched in eight N‐X‐S/T glycosylation motifs, a transmembrane region, and an intracellular tyrosine kinase domain [10]. FGFR1 activation is achieved by the binding of the FGF ligand and subsequent dimerization and conformational changes resulting in trans‐phosphorylation of tyrosine residues in the catalytic core of the receptor [11].

Whereas active FGFRs phosphorylate several adaptor proteins and transmit signals mostly from their primary localization, the plasma membrane, there have been multiple reports of the FGFRs' presence inside the cell, mainly in the mitochondria and nucleus. A fraction of FGFR1 was found in the mitochondria of lung cancer cells [12]. Although the mechanism of mitochondrial transport of FGFR1 is still unknown, it was shown that the mitochondrial fraction of FGFR1 stimulates the cancer cell growth by promoting energy generation via glycolysis (so‐called Warburg effect) [12]. Nuclear accumulation of FGFR1 was observed in numerous cell lines and was implicated in playing a role in the regulation of gene expression, integration of extracellular and intracellular signaling, modulation of the cell cycle, cell development, and survival [13, 14, 15, 16, 17, 18, 19, 20].

Our recent studies showed the link between glycosylation and subcellular localization of glycosylation‐deficient mutant of FGFR1 (FGFR1.GF) [21]. FGFR1.GF is an artificial variant whose properties largely resemble FGFR2 and FGFR3 disease‐linked mutants characterized by altered N‐glycosylation [21, 22, 23, 24, 25, 26]. FGFR1.GF accumulated in the nuclear envelope and endoplasmic reticulum (ER) and displayed a high level of ligand‐independent autoactivation [21]. FGFR1.GF is capable of FGF recognition, yet it does not respond to extracellular growth factors to the intracellular accumulation [21]. We proposed the model of the N‐glycosylation‐dependent cellular trafficking of FGFR1, where the N‐glycans of the D2 and D3 domains are essential for FGFR1 targeting to the plasma membrane and lack of N‐glycans results in FGFR1 retention in the nuclear envelope [21]. Recently, it was also proposed that increased intracellular retention of not fully glycosylated FGFRs is caused by ER stress [27].

Peroxisomes are single membrane‐bound, highly dynamic organelles implicated in diverse metabolic pathways [28, 29]. The biogenesis of peroxisomes occurs via two distinct pathways: growth and division of preexisting peroxisomes and de novo formation from the ER, ensuring maintenance of proper peroxisome numbers and allowing for rapid adaptation to metabolic needs [29]. The biogenesis of most peroxisomal membrane proteins starts within the ER [29, 30]. Next, pre‐peroxisomal vesicles loaded with peroxisomal membrane proteins bud from the ER and mature into functional peroxisomes [30]. Since FGFR1.GF accumulates in the ER, here we studied whether it can subsequently traffic from the ER to peroxisomes.

FGFR1.GF is characterized by a distinct set of partner proteins in comparison to the wild‐type FGFR1 [21]. Importantly, we identified dehydrogenase/reductase SDR family member 2 (DHRS2) as protein co‐purifying exclusively with SBP‐FGFR1.GF [21, 31]. DHRS2 is involved in lipid metabolism and redox homeostasis, modulating cell proliferation and migration, and may play a role as a tumor suppressor [32, 33]. Because DHRS2 was the only one on the mass spectrometry (MS) hit list for binding only with SBP‐FGFR1.GF (and not with the wild‐type FGFR1), we decided to further focus on DHRS2‐FGFR1.GF interplay.

## 2 Methods

### 2.1 Antibodies and Reagents

Antibodies and fluorescent reagents used in this study are summarized in Table S1.

### 2.2 Cells

Mouse embryo fibroblast cells (NIH3T3) were cultured in Dulbecco's modified Eagle's medium—DMEM (Thermo Fisher Scientific, Waltham, MA, USA). Human osteosarcoma cell line (U2OS) was obtained from American Type Culture Collection (ATCC, Manassas, VA, USA) and was cultured in DMEM high glucose medium containing stable glutamine and sodium pyruvate (Biowest, Nuaillé, France). Media were supplemented with 10% fetal bovine serum (FBS) (Thermo Fisher Scientific) and antibiotics (100 U/mL penicillin, 100 μg/mL streptomycin). Polyclonal U2OS cell lines stably transfected with pcDNA3.1 vector containing the sequence encoding SBP‐FGFR1 (U2OS‐SBP‐R1) and SBP‐FGFR1.GF (U2OS‐SBP‐R1.GF) were prepared as described previously [34]. Transient transfections of U2OS cells with pcDNA3.1 vectors containing sequences encoding DHRS2 variants (Gene Universal, Newark, DE, USA) were performed with FuGENE HD Transfection Reagent (Promega) according to the manufacturer's instructions. For U2OS‐SBP‐R1 and U2OS‐SBP‐R1.GF cells growth media were additionally supplemented with geneticin (1.5 mg/mL) (Thermo Fisher Scientific). All cells were cultivated in 5% CO_2_ atmosphere at 37°C and seeded into tissue culture plates 1 day prior to the start of the experiments.

### 2.3 Mass Spectrometry

Mass spectrometry experiments were prepared and performed as described before [21].

### 2.4 Proximity Ligation Assay (PLA)

To analyze the interactions between FGFR1 variants and DHRS2 or FGFR1.GF and PEX14, Duolink In Situ Fluorescence Protocol was used (Sigma‐Aldrich, St. Louis, Missouri, USA). U2OS‐SBP‐R1 and U2OS‐SBP‐R1.GF cells were fixed with 4% paraformaldehyde (PFA) and permeabilized with 0.1% Triton in Phosphate‐Buffered Saline (PBS). Cells were then incubated with mouse anti‐SBP tag and rabbit anti‐DHRS2 or rabbit PEX14 antibodies. Cells were then treated according to the manufacturer's protocols. Cell nuclei were stained with NucBlue Live dye. Fluorescence microscopy for the protein interactions was carried out using the Zeiss Axio Observer Z1 fluorescence microscope (Zeiss, Oberkochen, Germany) or the Opera Phenix Plus High‐Content Screening system (Perkin Elmer, Waltham, MA, USA). Image acquisition and analysis was carried out using the Zeiss ZEN 2.3 software (Zeiss, Oberkochen, Germany) or the Harmony High‐Content Imaging and Analysis Software (version 5.1; Perkin Elmer) and Adobe Photoshop (Adobe, San Jose, CA, USA).

### 2.5 Fluorescence Microscopy

Cells were washed with PBS, fixed with 4% PFA, permeabilized with 0.1% Triton in PBS, and blocked in 2% BSA in PBS. Incubation times for primary antibodies were either 2 h at room temperature (RT) or 18 h at 4°C, and for secondary antibodies 1 h at RT. Nuclei were labeled with a NucBlue Live dye, and cytoplasm was labeled using HCS CellMask Deep Red Stain.

For the mitochondria staining, the cells were incubated for 18 h with mitochondrial marker, CellLight Mitochondria‐RFP prior to fixing. For detection of FGFR1 in immunofluorescence microscopy experiments, high affinity, highly selective tetravalent anti‐FGFR1 T‐Fc antibody was used [35]. Cells were incubated with T‐Fc at 37°C for 1 h prior to blocking. T‐Fc was visualized using Zenon Human IgG Labeling Kit, and nuclei were labeled with a NucBlue Live dye.

For co‐localization of the PLA signals between FGFR1 and DHRS2 with mitochondria or peroxisomes, after the PLA experiment, cells were incubated with a mitochondrial marker, cytochrome c antibody, or peroxisomal marker, PEX14 antibody. Nuclei were labeled with a NucBlue Live dye and cytoplasm was labeled with HCS CellMask Deep Red Stain. The cytochrome c or PEX14 fluorescence signal intensity was used to determine the region of interest (ROI) for each organelle (mitochondria or peroxisomes). The fluorescence signal intensity of PLA was measured across the cell and then in the ROI for the organelle. The results were compared with each other, and co‐localization was calculated.

For analysis of peroxisome numbers, cells transfected with 20 nM nontargeting siRNA or 20 nM siRNA against DHRS2 were stained for PEX14 or PEX11β and cytoplasm (HCS CellMask Deep Red Stain) and imaged using the Opera Phenix Plus High‐Content Screening system (Perkin Elmer). The number of cells was determined using the Cell Mask Deep Red signal. The number of spots was determined based on PEX14 or PEX11β signal.

All confocal fluorescence microscopy measurements were carried out using the Opera Phenix Plus High‐Content Screening system (Perkin Elmer). Fixed cells were imaged in confocal mode using a 63 × Water, NA 1.15 objective with binning 2 using two peaks autofocus. Images were performed using a 2160 × 2160 px Camera ROI, 37 fields per well, with 8–10 Z‐stacks per field at 0.5 μm interval to ensure comprehensive imaging of the cell. The number of cells was determined using the DAPI signal, which enables nuclei detection, or the Cell Mask Deep Red signal, which enables cytoplasm detection. Images were assembled in Adobe Photoshop and Adobe Illustrator with only linear adjustments of contrast and brightness.

High‐resolution imaging was performed using a ZEISS Elyra 7 with a Lattice SIM^2^ super‐resolution microscope equipped with a 63 × 1.4 NA oil immersion Plan‐Apochromat objective at 30°C. Samples were illuminated using 488, 561, and 642 nm laser lines, and an LBF 405/488/561/642 dichroic mirror was inserted into the optical path. Fluorescent signals were collected using an sCMOS pco‐edge 4.2M camera. Raw images (13 phase‐shifted) were reconstructed using ZEN black software (ZEN 3.0 SR FP2; Carl Zeiss, Jena, Germany) with the SIM2 Lattice module.

### 2.6 siRNA Transfection

siRNA transfections were performed with DharmaFECT 1 Transfection Reagent according to the manufacturer's instructions. DharmaFECT 1 Transfection Reagent (#T‐2001‐02) and nontargeting siRNA (#D‐001810‐10‐20) were from Horizon (Cambridge, UK). siRNA against DHRS2 (#sc‐92 153) was from Santa Cruz Biotechnology (Dallas, TX, USA). Cells were transfected with 20 nM siRNA against DHRS2 or 20 nM nontargeting siRNA as a control. After 24 h, the transfection medium was replaced with the complete medium. Cells were incubated in a 5% CO_2_ atmosphere at 37°C for another 48 h.

### 2.7 Catalase Activity Assay

Catalase activity was measured using Catalase Colorimetric Activity Kit (#EIACATC) from Invitrogen (Carlsbad, CA, USA). U2OS, U2OS‐SBP‐R1, and U2OS‐SBP‐R1.GF cells were washed with PBS and resuspended in 1 mL of cold 1× Assay Buffer. Cells were then treated according to the manufacturer's protocols. Catalase activity (U/mL) was calculated by generating a four‐parameter logistic regression standard curve using the AAT Bioquest online resource, which was also used to convert absorbance values to activity values. The protein concentration in the samples was determined using Pierce Bradford Plus Protein Assay (Thermo Fisher Scientific). Catalase activity (U/μg of total protein) was calculated from the standard curve equation and normalized to protein concentration. Data were normalized to U2OS cells.

### 2.8 Recombinant Proteins

DHRS2.StrepTagII construct in pET‐3d vector allowing the expression of the protein was obtained via gene synthesis (Gene Universal, Newark, DE, USA). DHRS2.StrepTagII was expressed in *Escherichia coli* BL21 CodonPlus(DE3)‐RIL (Agilent Technologies, Santa Clara, CA, USA). Cells harboring the appropriate vectors were grown at 37°C until OD_600_ = 0.8. Protein expression was induced by addition of 0.1 mM IPTG, and then cells were incubated at 16°C overnight. Proteins were purified by affinity chromatography using Strep‐TactinXT 4Flow resin (#2‐5010‐010) and desthiobiotin (#2‐1000‐002) from IBA Lifesciences (Göttingen, Germany). The purity and the identity of obtained protein were confirmed by SDS‐PAGE and western blotting.

Glycosylated extracellular domain of FGFR1 fused to the Fc antibody fragment (FGFR1‐Fc) and his‐tagged kinase domain of FGFR1 (FGFR1‐KD) were prepared as described before [36, 37].

### 2.9 FGFR Activation

Serum‐starved NIH3T3 cells were stimulated for 15 min with increasing concentrations of DHRS2 (20 ng/mL, 200 ng/mL, 2 μg/mL, and 20 μg/mL) and FGF1 (20 ng/mL). Cells were lysed in Laemmli buffer and subjected to SDS‐PAGE and western blotting.

In the experiment with ligand trap (an extracellular region of FGFR1 fused to the Fc fragment of IgG1) serum‐starved NIH3T3 cells were preincubated with FGFR1‐Fc (10 μg/mL) for 1 h at 37°C in the presence of 10 U/mL heparin (Sigma‐Aldrich). Then, the cells were treated with DHRS2 (20 μg/mL) or FGF1 (20 ng/mL) for 15 min. The activation of signaling cascades was evaluated by western blotting.

### 2.10 Cell Proliferation

U2OS, U2OS‐SBP‐R1, and U2OS‐SBP‐R1.GF cells were seeded in DMEM with 10% FBS and antibiotics. Then, cells were incubated at 37°C, 5% CO_2_ for 72 h, and cell proliferation was determined with Presto Blue Cell Viability Reagent (Thermo Fisher Scientific).

### 2.11 Cell Migration

Cell migration was analyzed using the IncuCyte Cell Migration and Invasion System (Essen BioScience, Royston, UK). U2OS, U2OS‐SBP‐R1, and U2OS‐SBP‐R1.GF cells were seeded into a 96‐well IncuCyte ImageLock plate at a density of 3 × 10^4^ cells/well, in DMEM with 10% FBS and antibiotics and scratched with IncuCyte WoundMaker. Wound density was monitored over 50 h, with images automatically acquired every 2 h using an IncuCyte ZOOM 10× objective and then analyzed within the wound area using the IncuCyte ZOOM GUI Version: 2018A Software package.

### 2.12 Bio‐Layer Interferometry (BLI)

To analyze the interaction between FGFR1 variants and DHRS2, BLI measurements were conducted using the Octet RED K2 system (ForteBio, San Jose, CA, USA). DHRS2 (50 μg/mL) was immobilized in PBS on SAX sensors in a pairwise manner (studied protein and the reference sensor), and sensors were subsequently incubated with FGFR1‐Fc or FGFR1‐KD (50 μg/mL). As a control, no protein was immobilized on the SAX sensor, and subsequently, the interaction with FGFR1‐Fc or FGFR1.GF‐Fc (50 μg/mL) was analyzed. To study the impact of FGFR1 N‐glycosylation on the interaction with DHRS2, non‐treated FGFR1‐Fc and PNGase F‐deglycosylated FGFR1‐Fc (50 μg/mL) were immobilized on Protein‐A biosensors and incubated with DHRS2 (50 μg/mL). Subsequently, the interaction of DHRS2 with non‐treated FGFR1‐Fc and PNGase F‐deglycosylated FGFR1‐Fc was analyzed.

### 2.13 Deglycosylation

Deglycosylation of FGFR1‐Fc for the BLI experiments was performed in non‐denaturing reaction conditions using PNGase F (#P0704) from New England Biolabs (Ipswich, MA, USA) according to the manufacturer's instructions and established protocols [38]. Briefly, 7 μg of FGFR1‐Fc was incubated with 2 μL of PNGase F in 37°C for 1.5 h. Protein was then analyzed using BLI and western blotting.

### 2.14 Statistical Analysis

All experiments were performed in at least three independent biological replicates. Data are presented as mean ± standard deviation (SD). Statistical analysis was performed using Microsoft Excel (Microsoft Corporation) and GraphPad Prism (GraphPad Software). Normality for all data was assessed using a panel of D'Agostino & Pearson, Anderson‐Darling, Shapiro–Wilk, and Kolmogorov–Smirnov tests. Data were analyzed using robust regression (ROUT method, *Q* = 1%) to downweigh the influence of outliers. Significance levels were indicated as ns, not significant; *p < 0.05; **p < 0.005; ***p < 0.001; and ****p < 0.0001. Statistical significance was determined by Mann–Whitney test, Kruskal–Wallis test, or one‐way ANOVA with Tukey's post hoc test. The statistical tests used in respective experiments are described in the figure legends.

## 3 Results

### 3.1 DHRS2 Is a Dually Localized Protein

**FIGURE 1:**
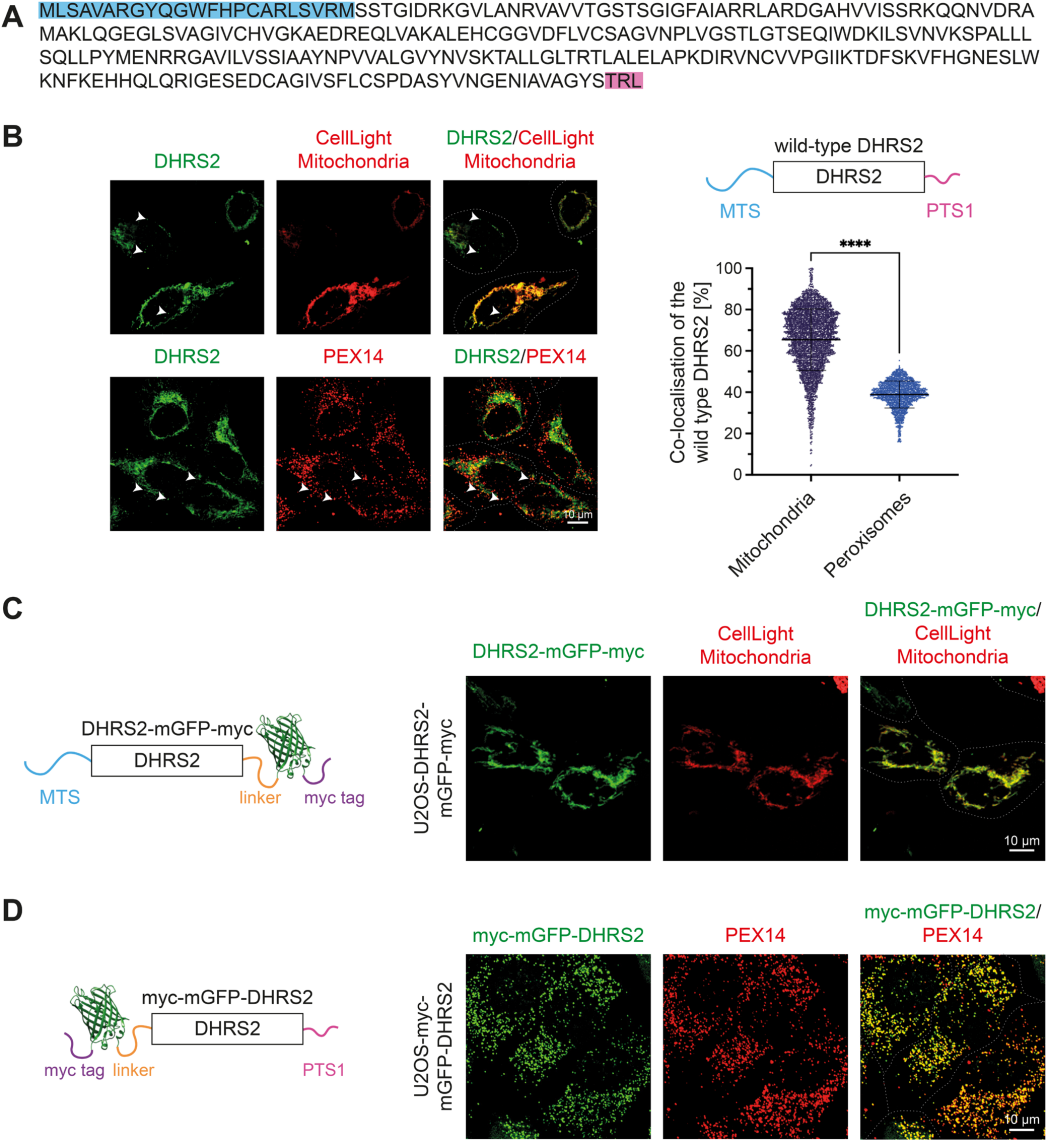
DHRS2 is a unique protein with two localization signal sequences. (A) Sequence of wild‐type DHRS2 (Uniprot ID Q13268) and its schematic representation. Mitochondrial targeting signal (MTS) predicted by TargetP and MitoProt II is highlighted in blue. These subcellular localization predictions showed 0.993 and 0.9053 probability of export to mitochondria, respectively. Peroxisomal targeting signal 1 (PTS1) predicted by The PTS1 Predictor using the general function with the score of 0.462 is highlighted in pink. (B) Immunofluorescence‐based co‐localization of wild‐type DHRS2 with the mitochondrial marker CellLight Mitochondria and the peroxisomal marker PEX14 in U2OS‐SBP‐R1.GF cells. Arrowheads indicate selected areas of signals co‐localization. Scale bar represents 10 μm. The estimated cell boundaries are marked with gray dashed lines. Schematic representation of the wild‐type DHRS2 and analysis of the co‐localisation are shown. Single dot represents the percentage of the co‐localization of the DHRS2 signal with the mitochondrial or peroxisomal markers recorded in individual cell. At least 3000 cells for each staining were analyzed. Statistical analyses were performed using Mann–Whitney test. Data are presented as mean ± SD. ns, not significant; ****p < 0.0001. (C) Immunofluorescence‐based co‐localization of DHRS2‐mGFP‐myc with mitochondrial marker (CellLight Mitochondria). Schematic representation of the tested DHRS2 localization variant is shown. Arrowheads indicate selected areas of signals co‐localization. The estimated cell boundaries are marked with gray dashed lines. Scale bar represents 10 μm. (D) Immunofluorescence‐based co‐localization of myc‐mGFP‐DHRS2 variant with the peroxisomal marker protein (PEX14). Schematic representation of the tested DHRS2 localization variant is shown. Arrowheads indicate selected areas of signals co‐localization. The estimated cell boundaries are marked with gray dashed lines. Scale bar represents 10 μm.

DHRS2 is an intracellular protein previously reported to localize to cytoplasm, nucleus, and mitochondria [32, 39, 40]. DHRS2 bears a canonical mitochondrial targeting presequence (MTS) at the N‐terminus, implicating its targeting to the mitochondrial matrix [31, 41]. Interestingly, we also observed a putative peroxisomal targeting sequence 1 (PTS1) (−TRL) at the C‐terminus of DHRS2 (Figure 1A). Using available tools for prediction of targeting signals in proteins, with TargetP and MitoProt II we predicted MTS at the N‐terminus of DHRS2 with 0.993 and 0.9053 score, respectively (Figure 1A, highlighted in blue) [42, 43]. Using the PTS1 Predictor tool we detected putative PTS1 at the C‐terminus of DHRS2 with the score of 0.462 (Figure 1A, highlighted in pink) [44].

To study the subcellular localization of the wild‐type DHRS2, we performed immunofluorescence microscopy experiments with anti‐DHRS2 antibodies in U2OS cells stably expressing SBP‐FGFR1.GF (Figure 1B, Figure S1). We detected two types of DHRS2 signals in U2OS‐SBP‐FGFR1.GF cells: elongated, mostly perinuclear structures that co‐localize with CellLight Mitochondria and numerous puncta co‐localizing with PEX14 (Figure 1B). Using high‐content Opera Phenix Plus confocal microscopy platform and immunofluorescence staining we confirmed that the majority of DHRS2 signal co‐localizes with the mitochondria‐specific dye (Figure 1B). These data indicate that DHRS2 is a dually localized protein with higher abundance in mitochondria and a minor population present in peroxisomes.

To test the functionality of both mitochondrial and peroxisomal localization signals within DHRS2, we prepared two constructs of DHRS2: one, DHRS2‐mGFP‐myc, with accessible MTS only, in which we replaced PTS1 with the flexible linker and mGFP‐myc, and the second one, myc‐mGFP‐DHRS2, with MTS substituted by mGFP‐myc and exposed C‐terminal PTS1 only (Figure 1C,D). U2OS cells were transfected with DHRS2 variants, and their localization was studied with confocal microscopy. The transfection efficacy was confirmed using western blotting (Figure S2). DHRS2‐mGFP‐myc lacking the PTS1 sequence virtually fully co‐localized with the CellLight Mitochondria signal, confirming functionality of the MTS of DHRS2 (Figure 1C). The myc‐mGFP‐DHRS2 construct lacking the MTS sequence fully co‐localized with the peroxisomal marker protein, PEX14, demonstrating that the C‐terminal‐TRL sequence constitutes bona fide PTS1 signal of DHRS2 (Figure 1D).

These data indicate that DHRS2 contains two functional targeting signals triggering DHRS2 transport to the mitochondrial matrix and peroxisomal lumen.

### 3.2 DHRS2 Affects Peroxisome Abundance

**FIGURE 2:**
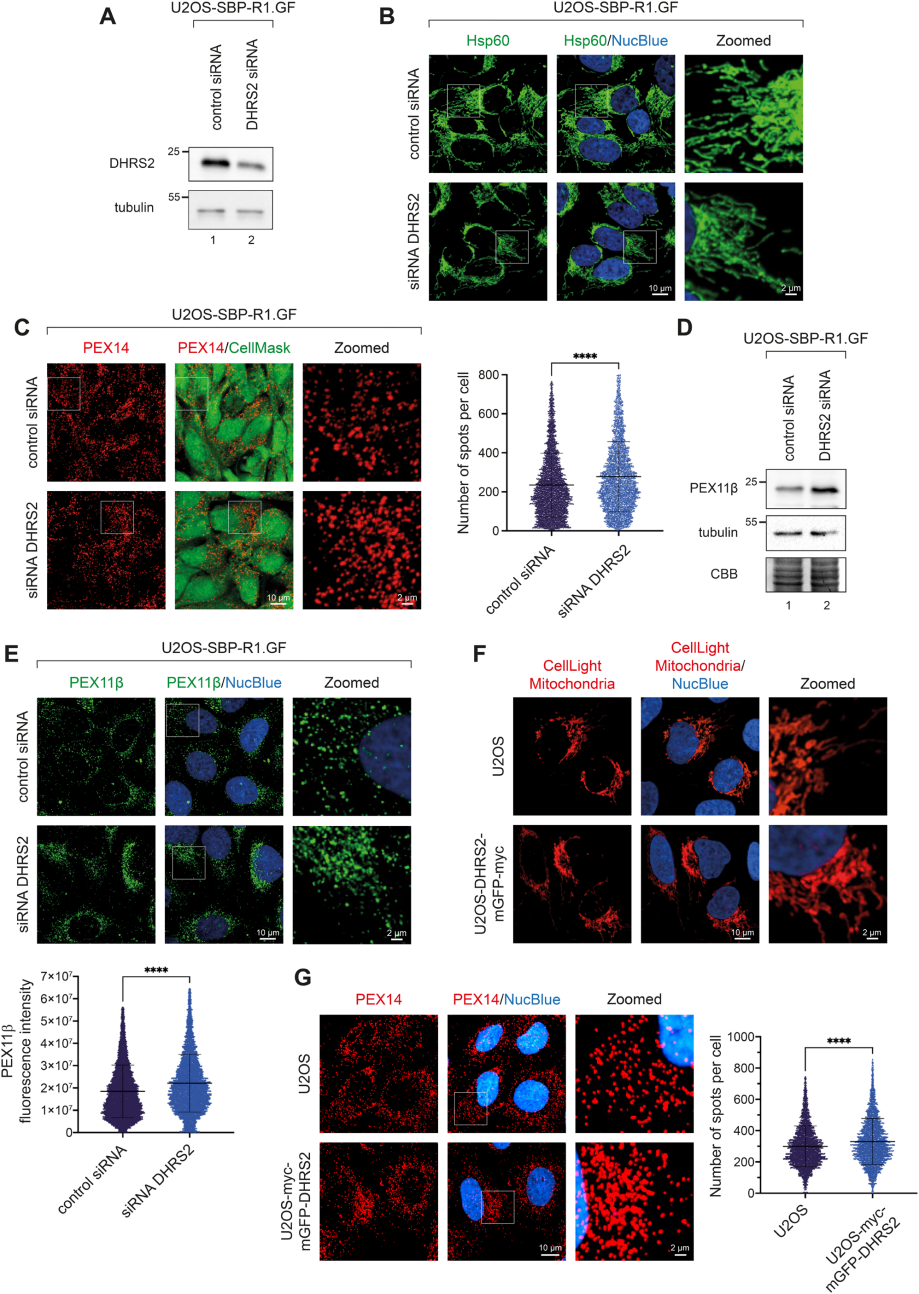
Impact of DHRS2 on the organelle morphology. (A) Western blotting analysis of cell lysates of U2OS‐SBP‐R1.GF cells treated with siRNA against DHRS2 and nontargeting siRNA as a control. (B) Impact of DHRS2 knock‐down on mitochondria morphology in U2OS‐SBP‐R1.GF cells. Hsp60 was used as a mitochondrial marker. Scale bar represents 10 or 2 μm for the zoomed fractions of the photos, respectively. (C) Impact of DHRS2 knock‐down on peroxisome morphology in U2OS‐SBP‐R1.GF cells analyzed using quantitative confocal microscopy. PEX14 was used as a peroxisomal marker. Single dot represents number of peroxisomes recorded in individual cell. Data shown in the graphs are mean PEX14 signal intensities ± SD. At least 4000 cells for each condition were analyzed. The estimated cell boundaries are marked with gray dashed lines. Scale bar represents 10 or 2 μm for the zoomed fractions of the photos, respectively. Statistical analyses were performed using Mann–Whitney test. Data are presented as mean ± SD. ns, not significant; ****p < 0.0001. (D) PEX11β levels in cell lysates of U2OS‐SBP‐R1.GF cells treated with DHRS2‐targeting or nontargeting siRNAs analyzed by western blotting. (E) Impact of DHRS2 knock‐down on peroxisome abundance in U2OS‐SBP‐R1.GF cells analyzed using quantitative confocal microscopy. PEX11β was used as a marker of peroxisomal biogenesis. Single dot represents fluorescence intensity recorded in individual cell. At least 4000 cells for each condition were analyzed. Scale bar represents 10 or 2 μm for the zoomed fractions of the photos, respectively. Statistical analyses were performed using Mann–Whitney test. Data are presented as mean ± SD. ns, not significant; ****p < 0.0001. (F) Impact of DHRS2‐mGFP‐myc on mitochondria morphology in transiently transfected U2OS cells (bottom panel). U2OS cells were stained as a control (top panel). Scale bar represents 10 μm or 2 μm for the zoomed fractions of the photos, respectively. (G) Impact of myc‐mGFP‐DHRS2on peroxisome morphology in transiently transfected U2OS cells (bottom panel). U2OS cells were stained as a control (top panel). Single dot represents number of peroxisomes recorded in individual cell. Data shown in the graphs are mean PEX14 signal intensities ± SD. At least 4000 cells for each condition were analyzed. Scale bar represents 10 or 2 μm for the zoomed fractions of the photos, respectively. Statistical analyses were performed using Mann–Whitney test. Data are presented as mean ± SD. ns, not significant; ****p < 0.0001.

To study the impact of DHRS2 on mitochondria and peroxisomes, we knocked down DHRS2 with siRNA (Figure 2A) and using confocal microscopy determined the impact of reduced DHRS2 level on mitochondrial morphology and peroxisome abundance. As shown in Figure 2B, depletion of DHRS2 had no impact on mitochondrial morphology. Using high‐content Opera Phenix Plus confocal microscopy platform and PEX14 immunofluorescence, we analyzed the abundance of peroxisomes in U2OS‐SBP‐FGFR1.GF cells upon downregulation of DHRS2. Knockdown of DHRS2 resulted in a significantly increased number of peroxisomes (Figure 2C).

Next, we analyzed if the increased number of peroxisomes upon DHRS2 silencing is accompanied by changes in the level of PEX11β, the master regulator of peroxisome division. Western blot analysis (Figure 2D) and quantitative immunofluorescence microscopy experiments (Figure 2E) revealed significantly increased PEX11β levels following DHRS2 knockdown.

We also studied the impact of overexpression of differentially localized DHRS2 variants on mitochondria and peroxisome morphology and abundance. As shown in Figure 2F,G, overexpression of DHRS2 variants had virtually no impact on mitochondrial and peroxisomal morphology. However, overexpression of the peroxisomal DHRS2 variant (myc‐mGFP‐DHRS2) resulted in a slightly increased number of peroxisomes (Figure 2G).

These results suggest that DHRS2 negatively regulates peroxisome biogenesis, affecting PEX11β levels. Furthermore, our data indicate that subcellular localization of DHRS2 is important for its effect on peroxisome numbers.

### 3.3 Glycosylation‐Deficient FGFR1.GF Interacts With DHRS2 in Peroxisomes

**FIGURE 3:**
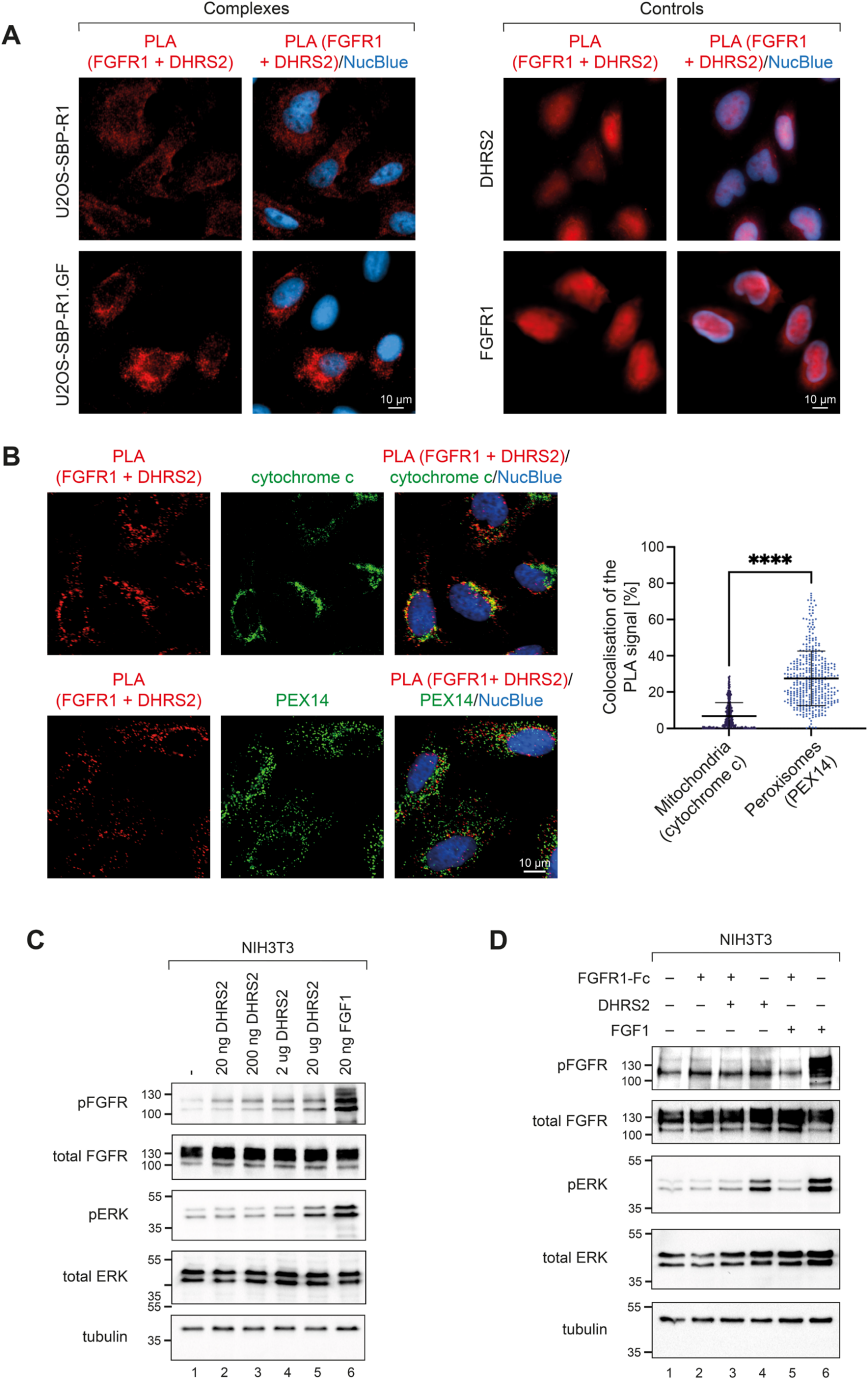
DHRS2 is a partner protein of FGFR1. (A) PLA confirmation of the interaction between SBP‐FGFR1.GF and DHRS2. Controls with single primary antibodies are shown. Scale bar represents 10 μm. (B) Immunofluorescence‐based co‐localization of PLA signals between SBP‐FGFR1.GF and DHRS2 with the mitochondrial marker protein cytochrome c and peroxisomal marker protein PEX14 in U2OS‐SBP‐R1.GF. Scale bar represents 10 μm. Single dot represents area of the co‐localization of the PLA signal with cytochrome c or PEX14 recorded in individual cell. Data shown in the graphs are mean fluorescence signal intensities ± SD. At least 400 cells for each staining were analyzed. Statistical analyses were performed using Mann–Whitney test. Data are presented as mean ± SD. ns, not significant; ****p < 0.0001. (C) Effects of DHRS2 on FGFR1 signaling. Serum‐starved NIH3T3 cells were treated with FGF1 (20 ng/mL, control) or increasing concentrations of recombinant DHRS2 (20 ng/mL–20 μg/mL). Cells were lysed and analyzed with western blotting. Tubulin served as a loading control. (D) Soluble extracellular FGFR1‐Fc blocks the activation of FGFR by DHRS2 in NIH3T3 cells. Cells were preincubated with FGFR1‐Fc (ligand trap; 10 μg/mL) for 1 h prior to simulation with DHRS2. FGF1 treatment was used as a control.

In order to confirm our previous mass spectrometry data suggesting FGFR1.GF‐DHRS2 interaction, we performed proximity ligation assay (PLA) tests. PLA experiments revealed specific intracellular PLA signals confirming FGFR1.GF interaction with DHRS2 (Figure 3A). The PLA signal in control U2OS‐SBP‐FGFR1 cells expressing the wild‐type, cell surface exposed N‐glycosylated FGFR1 was much lower than the one observed for U2OS‐SBP‐FGFR1 cells (Figure 3A), indicating that intracellular accumulation of FGFR1.GF promotes its interaction with DHRS2. Since DHRS2 is a dually localized protein, we decided to study in which subcellular compartment the FGFR1.GF/DHRS2 interaction takes place. To this end, we performed PLA experiments in U2OS‐SBP‐FGFR1.GF cells with concomitant staining of mitochondria (cytochrome c) or peroxisomes (PEX14) and measured co‐localization of PLA signals with specific organelle markers using quantitative confocal microscopy with Opera Phenix Plus HCS platform. As shown in Figure 3B, most of the PLA signal co‐localized with the peroxisome‐specific marker, indicating the proximity of FGFR1.GF with DHRS2 in peroxisomes.

To study if DHRS2 interaction with FGFR1.GF is direct, we produced recombinant DHRS2.StrepTag in a bacterial expression system (Figure S3A) and confirmed the identity of the purified protein with western blotting (Figure S3B). In the western blotting analyses, we observed multiple DHRS2.StrepTag bands, indicating that DHRS2.StrepTag forms oligomers (Figure S3B). Next, we decided to probe if DHRS2.StrepTag can directly interact either with the extracellular ligand binding domain of FGFR1 fused to the Fc fragment of IgG1, FGFR1‐Fc, or with the intracellular region of FGFR1 encompassing the tyrosine kinase domain, FGFR1‐KD, using bio‐layer interferometry (BLI). We immobilized DHRS2.StrepTag on SAX biosensors and subsequently incubated these biosensors with FGFR1‐KD; however, we observed no signal indicating interaction between tested proteins (Figure S4A). Similarly, we detected no interaction between FGFR1‐Fc and DHRS2.StrepTag (Figure S4B). Since we detected positive MS and PLA hits for glycosylation‐deficient FGFR1.GF and DHRS2, we enzymatically deglycosylated FGFR1‐Fc (Figure S4C), immobilized deglycosylated FGFR1‐Fc on Protein‐A biosensors, and incubated biosensors with DHRS2.StrepTag. Again, we detected no BLI signals (Figure S4D). Finally, we analyzed if recombinant DHRS2.StrepTag can activate cell surface FGFR1. The rationale behind this experiment was that if oligomeric DHRS2.StrepTag can interact with the extracellular part of FGFR1, it could promote FGFR1 clustering and activation in a similar manner to noncanonical FGFR1 ligands like galectins [10, 38]. Supplementation of serum‐starved NIH3T3 cells with increasing amounts of recombinant DHRS2.StrepTag resulted in a concentration‐dependent increase in FGFR1 phosphorylation and in the activation of FGFR1‐downstream ERK1/2; however, considerably high concentrations of DHRS2.StrepTag were required for FGFR1 activation (Figure 3C). To confirm the specificity of this effect, we performed signaling studies in the presence of recombinant soluble FGFR1‐Fc, acting as a ligand trap [45, 46]. Soluble FGFR1‐Fc blocked the FGF1‐ and DHRS2‐dependent activation of the cellular pool of FGFR and ERK1/2 (Figure 3D).

These data indicate that DHRS2 interacts with FGFR1.GF in peroxisomes. The extracellular region of FGFR1.GF seems important for the observed low affinity binding of DHRS2, or other cell surface components are additionally required for complex formation. Importantly, the discovery of DHRS2‐FGFR1.GF interplay implicated the possibility of FGFR1.GF targeting to peroxisomes.

### 3.4 A Minor Fraction of FGFR1.GF Is Localized to Peroxisomes

**FIGURE 4:**
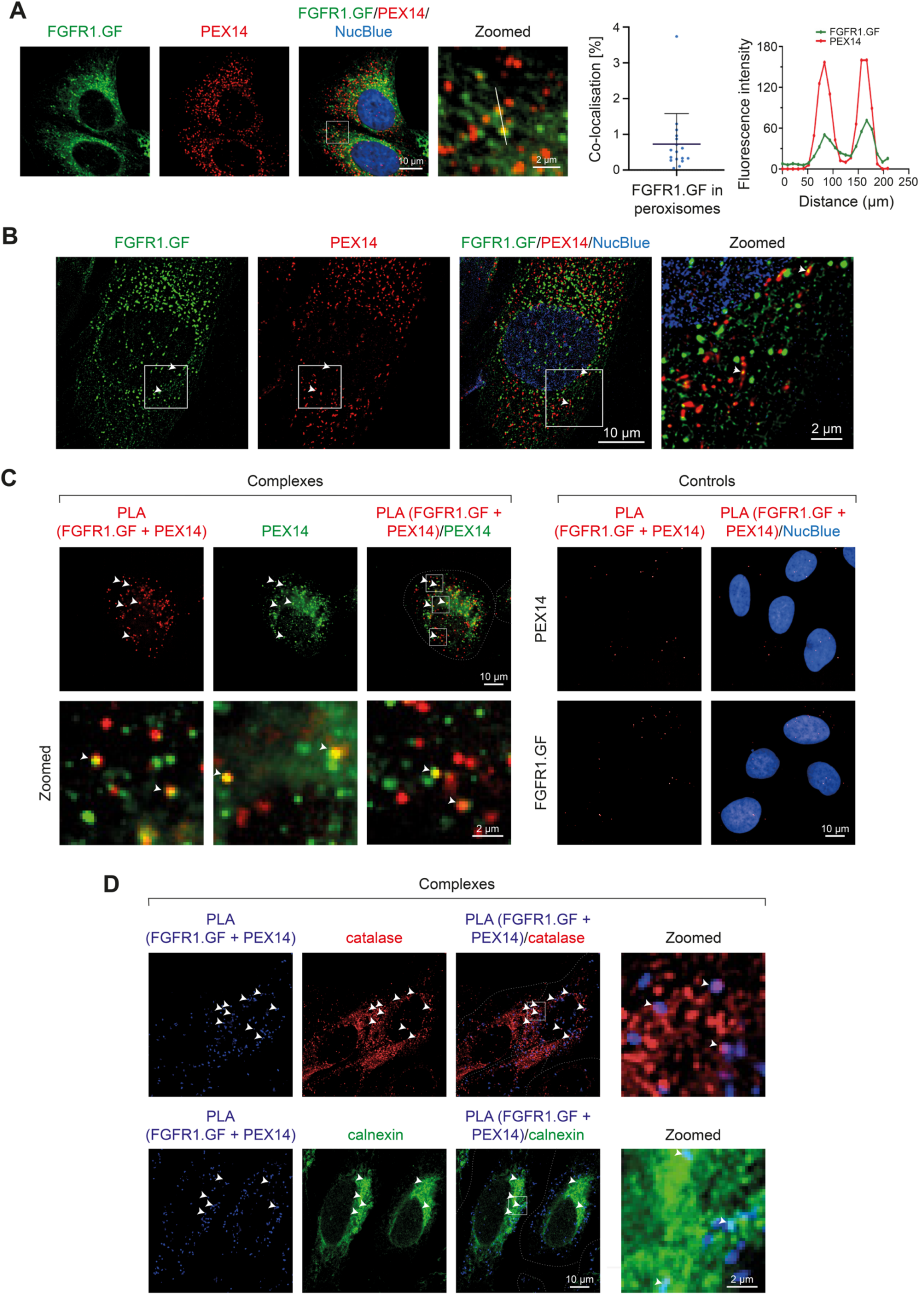
Fraction of FGFR1 is found in peroxisomes. (A) Co‐localization of SBP‐FGFR1 with PEX14 in U2OS‐SBP‐R1.GF cells analyzed by confocal fluorescence microscopy. Intensity profile of fluorescence of Alexa 488 (FGFR1.GF) and Alexa 594 (PEX14) is shown. Scale bar represents 10 or 2 μm for the zoomed fractions of the photos, respectively. (B) Co‐localization of SBP‐FGFR1 with PEX14 in U2OS‐SBP‐R1.GF cells visualized by super‐resolution imaging. Reconstructed SIM^2^ images are shown. 0.73% ± 0.85% of FGFR1 co‐localizes with PEX14 (mean ± SD, *n* = 17 cells). The analysis was prepared manually using the Fiji software. Scale bar represents 10 or 2 μm for the zoomed fractions of the photos, respectively. (C) Immunofluorescence‐based co‐localization of PLA‐based interaction between SBP‐FGFR1.GF and PEX14 with the peroxisomal marker protein PEX14 in U2OS‐SBP‐R1.GF cells. The bottom panel shows a magnified view of the highlighted area containing co‐localization of PLA signal with PEX14. Controls with single primary antibodies are shown. The estimated cell boundaries are marked with gray dashed lines. Scale bar represents 10 or 2 μm for the zoomed fractions of the photos, respectively. (D) Immunofluorescence‐based co‐localization of PLA‐based interaction between SBP‐FGFR1.GF and PEX14 with the ER marker calnexin and the peroxisomal lumen marker protein catalase in U2OS‐SBP‐R1.GF cells. The estimated cell boundaries are marked with gray dashed lines. Scale bars represent 10 or 2 μm for the zoomed fractions of the photos, respectively.

ER may serve as a source of peroxisomal membrane proteins, which are inserted into the ER membrane and after budding they are delivered to peroxisomes [30, 47]. Therefore, we wondered if FGFR1.GF that accumulates mostly in the ER could be partially delivered to peroxisomes, as indicated by above‐described PLA experiments. First, we analyzed co‐localization of SBP‐FGFR1.GF with PEX14 in U2OS‐SBP‐FGFR1.GF cells. Although the vast majority of SBP‐FGFR1.GF was found in the ER (Figure S5), we identified SBP‐FGFR1.GF punctate signal that occasionally co‐localized with PEX14 (Figure 4A, Figure S6). Confocal microscopy experiments revealed that about 1% of total FGFR1.GF co‐localized with PEX14 (Figure 4A). Additionally, using super‐resolution microscopy we confirmed this minor FGFR1.GF co‐localization with PEX14 (Figure 4B). Since FGFR1 is a transmembrane protein, we tested if FGFR1.GF is in proximity of model peroxisomal membrane protein PEX14. As shown in Figure 4C we detected positive PLA signals for FGFR1.GF‐PEX14 pair and these signals co‐localized with PEX14 immunofluorescence.

Biogenesis of peroxisomal membrane proteins may take place at the ER, from where pre‐peroxisomal vesicles may deliver membrane proteins to mature organelles, which are competent of peroxisome matrix protein import [30, 47]. To study if FGFR1.GF interaction with PEX14 takes place within the ER membranes, where FGFR1.GF accumulates, or is also found in mature peroxisomes, we studied co‐localization of PLA signals with calnexin (ER marker) and catalase (marker of matrix protein import‐competent peroxisomes). As demonstrated in Figure 4D, FGFR1.GF/PEX14 PLA signals co‐localized both with calnexin and with catalase.

Based on these findings, we hypothesize that a small fraction of ER‐trapped FGFR1.GF is transported to mature, import‐competent peroxisomes.

### 3.5 Overexpression of FGFR1 Variants Affects Peroxisome Abundance

**FIGURE 5:**
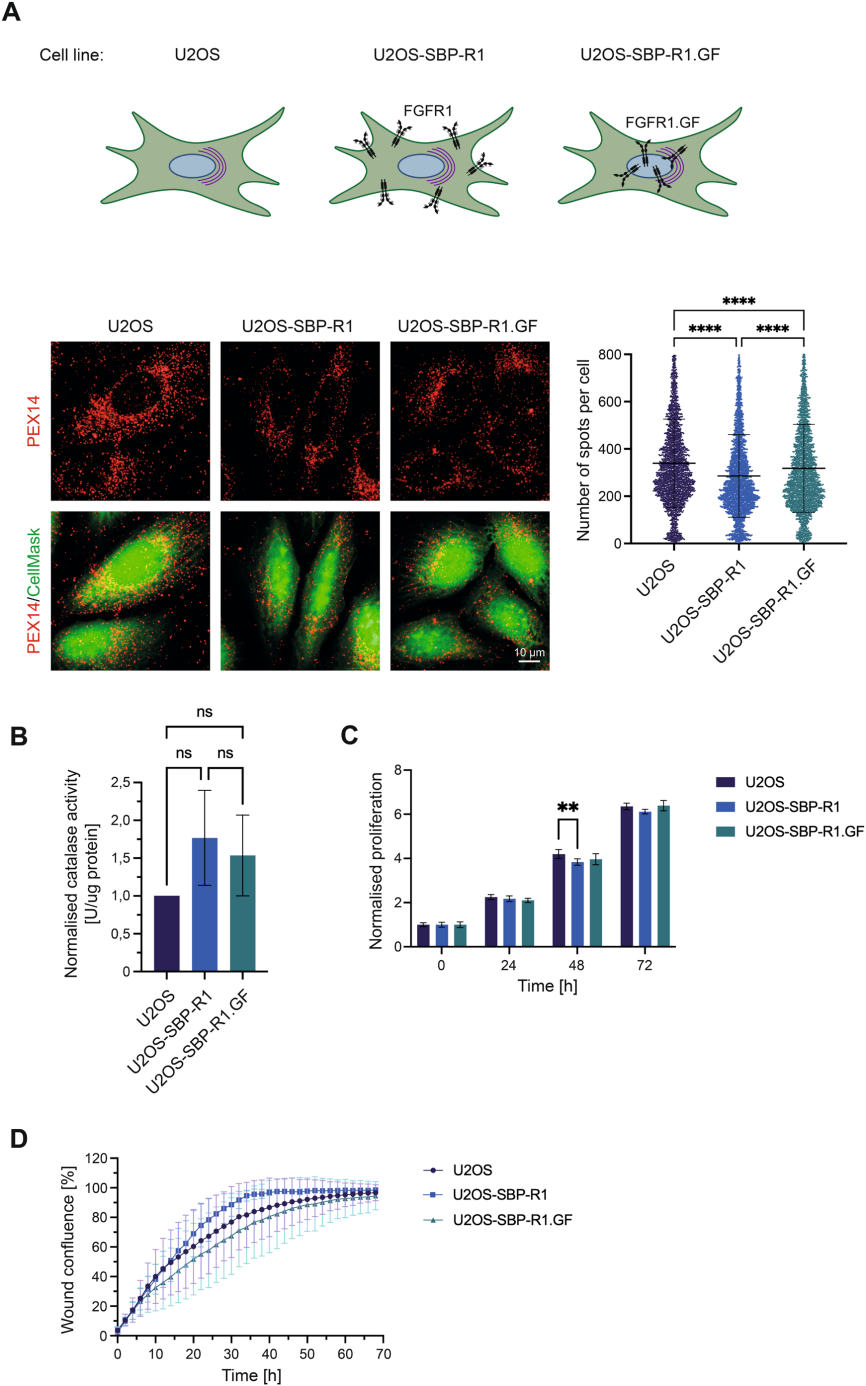
Impact of FGFR1 on peroxisome function and cell physiology. (A) U2OS, U2OS‐SBP‐R1, and U2OS‐SBP‐R1.GF cells were analyzed using quantitative confocal microscopy. Schematic representations of the used cell lines are shown. PEX14 was used as a peroxisomal marker. Single dot represents number of peroxisomes recorded in individual cell. At least 3300 cells per each cell line were analyzed. Scale bar represents 10 μm. The estimated cell boundaries are marked with gray dashed lines. Statistical analyses were performed using Kruskal–Wallis test. Data are presented as mean ± SD. ns, not significant; ****p < 0.0001. (B) Catalase activity in U2OS, U2OS‐SBP‐R1, and U2OS‐SBP‐R1.GF cells. Catalase activity was normalized to protein concentration (U/μg of total protein) and expressed relative to U2OS cells. Statistical analyses were performed using one‐way ANOVA with post hoc correction. Data are presented as mean ± SD. ns, not significant. (C) Cell proliferation of U2OS, U2OS‐SBP‐R1, and U2OS‐SBP‐R1.GF cells. Obtained results were normalized to U2OS cells. Statistical analyses were performed using one‐way ANOVA with post hoc correction. Data are presented as mean ± SD. ns, not significant; **p < 0.01. (D) Migratory capacity of U2OS, U2OS‐SBP‐R1, and U2OS‐SBP‐R1.GF cells. Cells were seeded onto ImageLock 96 well plates and scratched with IncuCyte WoundMaker. The rate of scratch closure was monitored for 70 h using the IncuCyte Cell Migration and Invasion System. Data are presented as a mean ± SD percentage representing the wound area occupied by migrating cells over time.

To study whether the wild‐type FGFR1 or glycosylation‐deficient FGFR1 variant have an impact on peroxisome abundance, using high‐content Opera Phenix confocal microscopy system we measured the number of peroxisomes in U2OS cells overproducing the wild‐type SBP‐FGFR1 and SBP‐FGFR1.GF. We observed a significantly lower number of peroxisomes in U2OS cells overproducing the wild‐type, plasma membrane‐targeted SBP‐FGFR1 in relation to U2OS cells with undetectable FGFR1 expression (Figure 5A). Interestingly, the number of peroxisomes in U2OS‐SBP‐FGFR1.GF was restored virtually to the level observed in U2OS cells (Figure 5A).

To evaluate the effect of the wild‐type FGFR1 and the glycosylation‐deficient FGFR1 variant on peroxisome function, we performed a catalase activity assay on U2OS cells that overproduce SBP‐FGFR1 or SBP‐FGFR1.GF. No significant changes in catalase activity were observed between U2OS‐SBP‐FGFR1 and U2OS‐SBP‐FGFR1.GF cells, or in comparison with control U2OS cells (Figure 5B).

To assess whether the wild‐type FGFR1 or the glycosylation‐deficient FGFR1 variant affects cell physiology, we performed proliferation and migration assays (Figure 5C,D). No detectable differences in proliferation rates or migratory potential between untreated U2OS‐SBP‐FGFR1 and U2OS‐SBP‐FGFR1.GF cells, or between U2OS‐SBP‐FGFR1.GF cells in relation to control U2OS cells were detected (Figure 4C,D).

Our data indicate that FGFR1, depending on subcellular localization, can affect peroxisome abundance.

## 4 Discussion

FGFR1 is typically localized on the cell surface, where it responds to the extracellular fibroblast growth factors, transmitting the signals across the plasma membrane [1]. Diminished FGFR1 N‐glycosylation (FGFR1.GF), which is observed for other FGFRs in Crouzon‐syndrome patients, predominantly in FGFR2 mutants, causes inhibition of FGFR transport to the cell surface, resulting in the accumulation of FGFR in the ER that finally leads to the transport of FGFR pool to the nuclear envelope [21, 22, 23]. The precise function and fate of intracellular ER/nuclear envelope‐trapped FGFRs is unknown [21]. Therefore, in our previous study we searched for binding partners of FGFR1.GF and among identified proteins we found DHRS2, which we followed in this study.

DHRS2 was reported to contain an N‐terminal mitochondrial targeting sequence that drives DHRS2 import into the mitochondrial matrix [31]. We also identified a C‐terminal PTS1 within the DHRS2 sequence and demonstrated that both MTS and PTS1 are active, driving the transport of DHRS2 into mitochondria and peroxisomes, respectively, and making DHRS2 a novel dually localized protein. Currently, it is unknown how the shuttling of DHRS2 between mitochondria and peroxisomes is regulated. It is tempting to speculate that posttranslational modifications of MTS and PTS1 may play a critical role in this process.

Since FGFR1 was reported to partially localize to mitochondria, we initially expected that MS‐indicated FGFR1.GF/DHRS2 complex would be predominantly localized to mitochondria. However, keeping in mind the revealed dual localization of DHRS2 to mitochondria and peroxisomes, we studied co‐localization of FGFR1.GF/DHRS2 PLA signals with mitochondria and peroxisomes. Unexpectedly, we revealed that FGFR1.GF/DHRS2 complexes were found in peroxisomes, indicating that the small fraction of FGFR1.GF is transported to these organelles. In agreement, we were able to co‐localize a small fraction of FGFR1.GF‐specific punctate signal with peroxisome‐specific marker PEX14. Using PLA, we demonstrated proximity between FGFR1.GF and PEX14 in peroxisomes. All these data support the conclusion that a minor pool of FGFR1.GF can reach peroxisomes. Yet, it remains to be elucidated if this FGFR1.GF pool is kinase‐active and whether it phosphorylates peroxisomal proteins.

**FIGURE 6:**
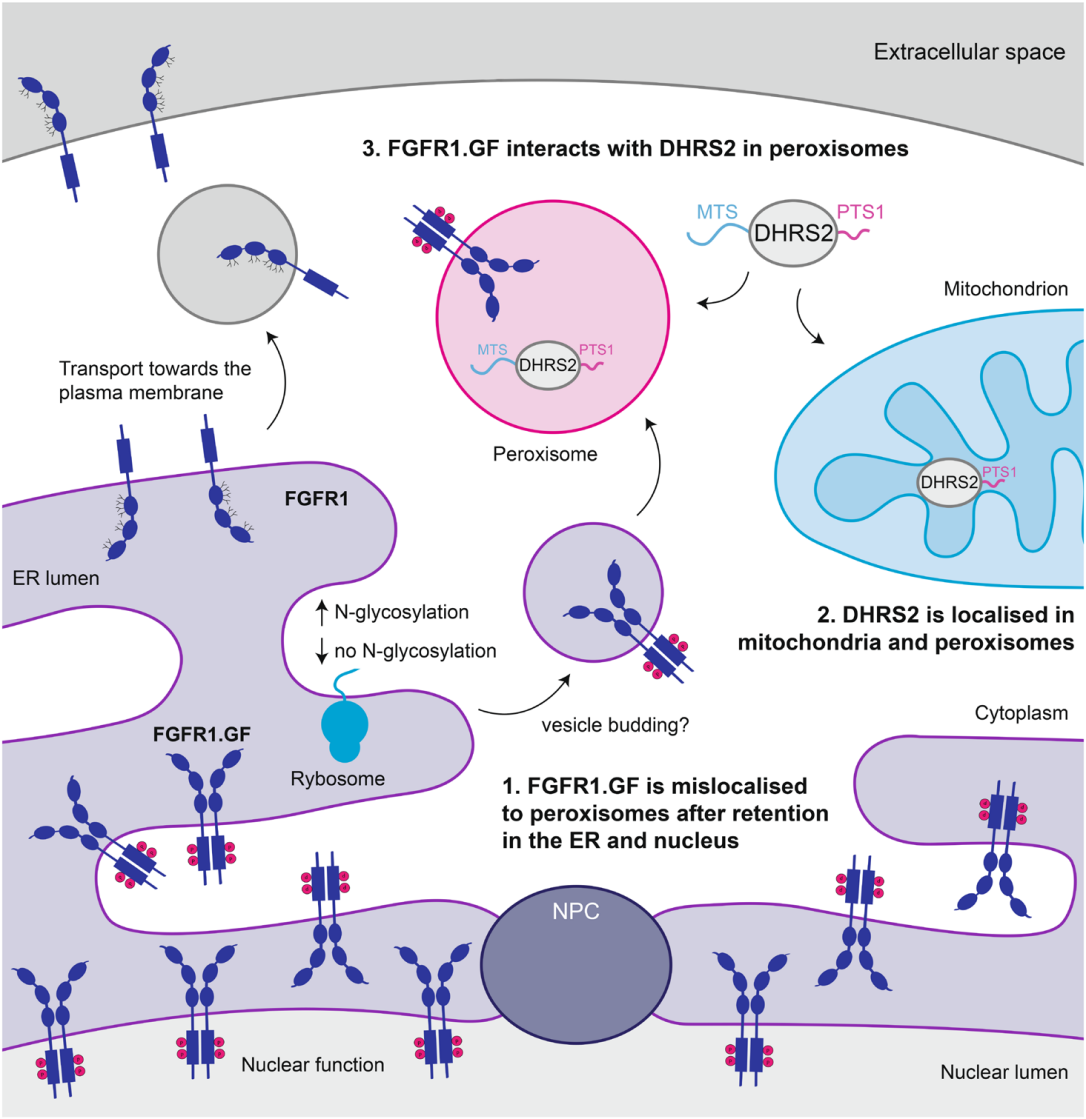
Hypothetical model of the N‐glycosylation‐dependent cellular trafficking of FGFR1. FGFR1 is co‐translationally inserted into ER membranes and N‐glycosylated at several positions, which is critical for FGFR1 progression through the secretory pathway and its final transport to the cell surface. Hampered N‐glycosylation (FGFR1.GF) leads to the intracellular accumulation of FGFR1 predominantly in the ER and in the nuclear envelope. Vesicles that transport peroxisomal membrane proteins from the ER to peroxisomes occasionally include FGFR1.GF that is highly abundant in the ER and deliver FGFR1 to peroxisomes. DHRS2 contains two targeting signal sequences, MTS and PTS1, that drive DHRS2 import into the mitochondrial matrix and peroxisome lumen, respectively. Peroxisomal fraction of DHRS2 rather indirectly interacts with peroxisomal fraction of FGFR1, allowing for visualization of FGFR1 in peroxisomes.

How FGFR1.GF can reach peroxisomes? Peroxisomes were for long considered as semiautonomous organelles capable of posttranslational import of matrix and membrane proteins directly from cytosol, and under specific conditions able to form de novo from the ER membranes [48]. However, it was later demonstrated that peroxisomal membrane proteins are inserted into the ER membranes, bud off from the ER and are delivered to peroxisomes in the vesicular form [30, 47, 49, 50, 51, 52]. We propose that an intracellular accumulation of FGFR1.GF in the ER membranes causes an occasional packing of FGFR1.GF into vesicles predestined to peroxisomes (Figure 6). To our knowledge, our data constitute the first report of RTK localized to peroxisomes. Taking into account the recent data showing RTK accumulation in the ER upon ER stress, it is highly probable that other RTKs can employ the mechanism proposed by us to reach peroxisomes [27].

The role of FGFR1 and DHRS2 in peroxisome biogenesis and function is unknown. Here, we demonstrate that changes in the level of DHRS2 are associated with alterations in peroxisome number, which is accompanied by altered PEX11β level. How cells modulate abundance of PEX11β in response to downregulation of DHRS2 is unknown. Since DHRS2 is mostly found in mitochondria and overexpression of the peroxisome‐targeted variant of DHRS2 caused increased peroxisome number, it is likely that mitochondrial DHRS2 by yet unknown mechanism suppresses the expression of PEX11β, affecting peroxisome abundance. The impact of FGFR1 on peroxisomes seems to be localization dependent. Overproduction of the wild‐type FGFR1, but not intracellular FGFR1.GF causes a decrease in peroxisome numbers, indicating that FGFR1 signals from the plasma membrane might play a role in peroxisome homeostasis. Clearly, further studies are required to reveal mechanisms of observed phenomena, especially in the context of oncogenic and anti‐oncogenic activities of FGFR1 and DHRS2, respectively. FGFR1 is a well‐studied oncogene and an emerging molecular target for selective treatment of diverse cancers [53]. In contrast, DHRS2 can act as a tumor suppressor, blocking cancer cell growth and metastasis [54, 55, 56, 57, 58, 59, 60, 61]. Our binding experiments with recombinant proteins could not provide strong evidence for a direct interaction between FGFR1.GF and DHRS2. However, the fact that extracellularly administered recombinant DHRS2 at high concentrations can activate FGFR1 suggests that a low affinity interaction between the extracellular domain of FGFR1 and DHRS2 is possible. Although only about 1% of FGFR1.GF reaches peroxisomes where a portion of DHRS2 is localized, the increased local concentrations of these proteins in the restricted volume of peroxisomes may promote their interaction. Alternatively, FGFR1.GF may interact with DHRS2 in an indirect manner via yet unknown proteins acting as a molecular bridge, whose absence in in vitro binding tests prevents detection of FGFR1/DHRS2 interaction. FGFR1 is a single spanning transmembrane protein, whose extracellular regions will be found in the lumen of intracellular organelles and vesicles (Figure 6). Such membrane topology allows for the interaction between peroxisomal FGFR1.GF and DHRS2 found in the peroxisomal matrix due to PTS1‐dependent import (Figure 6). The revealed by us functional connection between DHRS2 and FGFR1 requires further functional studies, especially in the context of tumor development and progression.

## Author Contributions

Conceptualization: Ł.O. and P.D.; methodology: all authors; software: P.D.; validation: all authors; formal analysis: all authors; investigation: P.D., A.C., and M.B.; resources: Ł.O.; data curation: all authors; writing – original draft: P.D. and Ł.O.; writing – review and editing: all authors; visualization: P.D., A.C., and Ł.O.; supervision: Ł.O. and N.P.; project administration: Ł.O.; funding acquisition: Ł.O.

## Funding

This work was supported by SONATA BIS grant (2019/34/E/NZ3/00014) from the National Science Centre awarded to Ł.O. The work of A.C., N.P., and Ł.O. was supported by the “PROT‐TOWING” grant (FENG.02.02‐IP.05‐0015) founded by the European Union within the First TEAM FENG action of the Foundation for Polish Science. The research collaboration was supported by NAWA Polish‐Chinese Common Research Projects Programme (BPN/BCH/2025/1/00007/U/00001) and Intergovernmental International Science and Technology Innovation Cooperation Key Project of China (2026YFE0153800).

## Conflicts of Interest

The authors declare no conflicts of interest.

## Supporting information


**Figure S1:** Panoramic view of the imaging of the immunofluorescence‐based co‐localization of wild‐type DHRS2 with the mitochondrial marker hsp60 and the peroxisomal marker protein PEX14 in U2OS‐SBP‐R1.GF cells. Scale bar represents 50 μm.
**Figure S2:** Western blot confirmation of U2OS cell line transfection with DHRS2‐mGFP‐myc and myc‐mGFP‐DHRS2 using myc antibody. The asterisk indicates the endogenous myc protein.
**Figure S3:** SDS‐PAGE analysis of purification of recombinant DHRS2 with C‐terminal StrepTagII from *E. coli* . DHRS2‐StrepTagII was purified using affinity chromatography. (A) Fractions collected during purification were resolved on SDS‐PAGE gel and visualized by Coomassie staining. Lane 1, molecular weight marker (kDa); Lane 2, cell pellet without induction (−IPTG); Lane 3, cell pellet with induction (+IPTG); Lane 4, insoluble fraction; Lane 5, soluble fraction; Lane 6, flow‐through; Lane 7, eluted fraction; Lane 8, fraction of the eluted protein after buffer exchange. The asterisk marks the target protein. (B) Confirmation of identity of DHRS2.StrepTagII using western blotting. Putative oligomeric forms of DHRS2.StrepTagII are marked with asterisks.
**Figure S4:** (A) BLI analysis of the interaction between DHRS2 and kinase domain of FGFR1 (FGFR1‐KD). DHRS2 was immobilized on SAX biosensors and incubated with equal mass concentrations of recombinant FGFR1‐KD to record the association and dissociation phases. Empty sensor control values were subtracted from the signal obtained for FGFRs. Representative results from at least three independent experiments are shown. (B) BLI analysis of the interaction between DHRS2 and extracellular domain of FGFR1 fused to Fc antibody fragment (FGFR1‐Fc). DHRS2 was immobilized on SAX biosensors and incubated with equal mass concentrations of recombinant FGFR1‐Fc to record the association and dissociation phases. Empty sensor control values were subtracted from the signal obtained for FGFR1. Representative results from at least three independent experiments are shown. (C) Western blot confirmation of FGFR1‐Fc deglycosylation after treatment with PNGase F. FGFR1‐Fc was detected using anti‐Fc antibody. The arrows indicate the mass shift. (D) BLI analysis of the interaction between DHRS2 and PNGase F‐deglycosylated FGFR1‐Fc. PNGase F‐deglycosylated FGFR1‐Fc was immobilized on Protein‐A biosensors and incubated with equal mass concentrations of DHRS2 to record the association and dissociation phases. Empty sensor control values were subtracted from the signal obtained for FGFR1.
**Figure S5:** Co‐localization of SBP‐FGFR1.GF with calnexin (ER marker) in U2OS‐SBP‐R1.GF cells and its analysis using quantitative confocal microscopy. Scale bar represents 10 or 2 μm for the zoomed fractions of the photos, respectively. Single dot represents co‐localization percentage calculated in individual cell. At least 800 cells were analyzed.
**Figure S6:** Co‐localization of SBP‐FGFR1.GF with PEX14 in U2OS‐SBP‐R1.GF cells. Scale bar represents 10 or 2 μm for the zoomed fractions of the photos, respectively.
**Table S1:** Summary of antibodies and fluorescent reagents used in this study. IF, immunofluorescence; PLA, proximity ligation assay; WB, western blotting.

## Data Availability

Data are available from the corresponding author upon request.

## References

[1] Y. Xie , N. Su , J. Yang , et al., “FGF/FGFR Signaling in Health and Disease,” Signal Transduction and Targeted Therapy 5 (2020): 181, 10.1038/s41392-020-00222-7. 32879300 PMC7468161

[2] D. M. Ornitz and N. Itoh , “New Developments in the Biology of Fibroblast Growth Factors,” WIREs Mechanisms of Disease 14 (2022): e1549, 10.1002/wsbm.1549. 35142107 PMC10115509

[3] Z. Wang and K. S. Anderson , “Therapeutic Targeting of FGFR Signaling in Head and Neck Cancer,” Cancer Journal 28 (2022): 354–362, 10.1097/PPO.0000000000000615. 36165723 PMC9523489

[4] S. Chen , Y. Qiu , P. Guo , T. Pu , Y. Feng , and H. Bu , “FGFR1 and HER1 or HER2 Co‐Amplification in Breast Cancer Indicate Poor Prognosis,” Oncology Letters 15 (2018): 8206–8214, 10.3892/ol.2018.8423. 29805554 PMC5950032

[5] O. Bogatyrova , J. S. M. Mattsson , E. M. Ross , et al., “FGFR1 Overexpression in Non‐Small Cell Lung Cancer Is Mediated by Genetic and Epigenetic Mechanisms and Is a Determinant of FGFR1 Inhibitor Response,” European Journal of Cancer 151 (2021): 136–149, 10.1016/j.ejca.2021.04.005. 33984662

[6] S. Mouron , L. Manso , E. Caleiras , et al., “FGFR1 Amplification or Overexpression and Hormonal Resistance in Luminal Breast Cancer: Rationale for a Triple Blockade of ER, CDK4/6, and FGFR1,” Breast Cancer Research 23 (2021): 21, 10.1186/s13058-021-01398-8. 33579347 PMC7881584

[7] H. U. Schildhaus , L. Nogova , J. Wolf , and R. Buettner , “FGFR1 Amplifications in Squamous Cell Carcinomas of the Lung: Diagnostic and Therapeutic Implications,” Translational Lung Cancer Research 2 (2013): 92–100, 10.3978/j.issn.2218-6751.2013.03.03. 25806220 PMC4369858

[8] M. A. Krook , J. W. Reeser , G. Ernst , et al., “Fibroblast Growth Factor Receptors in Cancer: Genetic Alterations, Diagnostics, Therapeutic Targets and Mechanisms of Resistance,” British Journal of Cancer 124 (2021): 880–892, 10.1038/S41416-020-01157-0. 33268819 PMC7921129

[9] P. Zhang , L. Yue , Q. Q. Leng , et al., “Targeting FGFR for Cancer Therapy,” Journal of Hematology & Oncology 17 (2024): 39, 10.1186/s13045-024-01558-1. 38831455 PMC11149307

[10] A. Gędaj , P. Gregorczyk , D. Żukowska , et al., “Glycosylation of FGF/FGFR: An Underrated Sweet Code Regulating Cellular Signaling Programs,” Cytokine & Growth Factor Reviews 77 (2024): 39–55, 10.1016/J.CYTOGFR.2024.04.001. 38719671

[11] B. Farrell and A. L. Breeze , “Structure, Activation and Dysregulation of Fibroblast Growth Factor Receptor Kinases: Perspectives for Clinical Targeting,” Biochemical Society Transactions 46 (2018): 1753–1770, 10.1042/BST20180004. 30545934 PMC6299260

[12] T. Hitosugi , J. Fan , T. W. Chung , et al., “Tyrosine Phosphorylation of Mitochondrial Pyruvate Dehydrogenase Kinase 1 Is Important for Cancer Metabolism,” Molecular Cell 44 (2011): 864–877, 10.1016/j.molcel.2011.10.015. 22195962 PMC3246218

[13] S. J. Coleman , A. M. Chioni , M. Ghallab , et al., “Nuclear Translocation of FGFR1 and FGF2 in Pancreatic Stellate Cells Facilitates Pancreatic Cancer Cell Invasion,” EMBO Molecular Medicine 6 (2014): 467–481, 10.1002/EMMM.201302698. 24503018 PMC3992074

[14] Y. Gao , Y. Wang , J. Yu , and R. Guo , “FGF Exhibits an Important Biological Role on Regulating Cell Proliferation of Breast Cancer When It Transports Into the Cell Nuclei,” Cell Biochemistry and Biophysics 80 (2022): 311–320, 10.1007/s12013-021-01044-2. 34796419

[15] A. Servetto , R. Kollipara , L. Formisano , et al., “Nuclear FGFR1 Regulates Gene Transcription and Promotes Antiestrogen Resistance in ER+ Breast Cancer,” Clinical Cancer Research 27 (2021): 4379–4396, 10.1158/1078-0432.CCR-20-3905. 34011560 PMC8338892

[16] I. Prudovsky , N. Savion , X. Zhan , et al., “Intact and Functional Fibroblast Growth Factor (FGF) Receptor‐1 Trafficks Near the Nucleus in Response to FGF‐1,” Journal of Biological Chemistry 269 (1994): 31720–31724, 10.1016/s0021-9258(18)31755-1. 7527394

[17] J. F. Reilly , E. Mizukoshi , and P. A. Maher , “Ligand Dependent and Independent Internalization and Nuclear Translocation of Fibroblast Growth Factor (FGF) Receptor 1,” DNA and Cell Biology 23 (2004): 538–548, 10.1089/dna.2004.23.538. 15383174

[18] P. A. Maher , “Nuclear Translocation of Fibroblast Growth Factor (FGF) Receptors in Response to FGF‐2,” Journal of Cell Biology 134 (1996): 529–536, 10.1083/jcb.134.2.529. 8707835 PMC2120872

[19] E. K. Stachowiak , P. A. Maher , J. Tucholski , et al., “Nuclear Accumulation of Fibroblast Growth Factor Receptors in Human Glial Cells–Association With Cell Proliferation,” Oncogene 14 (1997): 2201–2211, 10.1038/SJ.ONC.1201057. 9174056

[20] M. K. Stachowiak , P. A. Maher , A. Joy , E. Mordechai , and E. K. Stachowiak , “Nuclear Accumulation of Fibroblast Growth Factor Receptors Is Regulated by Multiple Signals in Adrenal Medullary Cells,” Molecular Biology of the Cell 7 (1996): 1299–1317, 10.1091/mbc.7.8.1299. 8856671 PMC275979

[21] P. Gregorczyk , N. Porębska , D. Żukowska , et al., “N‐Glycosylation Acts as a Switch for FGFR1 Trafficking Between the Plasma Membrane and Nuclear Envelope,” Cell Communication and Signaling 21 (2023): 177, 10.1186/s12964-023-01203-3. 37480072 PMC10362638

[22] N. E. Hatch , M. Hudson , M. L. Seto , M. L. Cunningham , and M. Bothwell , “Intracellular Retention, Degradation, and Signaling of Glycosylation‐Deficient FGFR2 and Craniosynostosis Syndrome‐Associated FGFR2C278F,” Journal of Biological Chemistry 281 (2006): 27292–27305, 10.1074/jbc.M600448200. 16844695

[23] U. Hashimoto , N. Fujitani , Y. Uehara , et al., “N‐Glycan on N262 of FGFR3 Regulates the Intracellular Localization and Phosphorylation of the Receptor,” Biochimica et Biophysica Acta (BBA)—General Subjects 1868 (2024): 130565, 10.1016/j.bbagen.2024.130565. 38244702

[24] C. Zammit , R. Barnard , J. Gomm , et al., “Altered Intracellular Localization of Fibroblast Growth Factor Receptor 3 in Human Breast Cancer,” Journal of Pathology 194 (2001): 27–34, 10.1002/PATH.846. 11329138

[25] C. L. Johnston , H. C. Cox , J. J. Gomm , and R. C. Coombes , “Fibroblast Growth Factor Receptors (FGFRs) Localize in Different Cellular Compartments: A Splice Variant of FGFR‐3 Localizes to the Nucleus,” Journal of Biological Chemistry 270 (1995): 30643–30650, 10.1074/JBC.270.51.30643. 8530501

[26] A. L. Delezoide , C. Lasselin‐Benoist , L. Legeai‐Mallet , et al., “Abnormal FGFR 3 Expression in Cartilage of Thanatophoric Dysplasia Fetuses,” Human Molecular Genetics 6 (1997): 1899–1906, 10.1093/hmg/6.11.1899. 9302269

[27] M. Bosakova , S. P. Abraham , D. Wachtell , et al., “Endoplasmic Reticulum Stress Disrupts Signaling via Altered Processing of Transmembrane Receptors,” Cell Communication and Signaling: CCS 23 (2025): 209, 10.1186/s12964-025-02208-w. 40307870 PMC12044870

[28] R. Kumar , M. Islinger , H. Worthy , R. Carmichael , and M. Schrader , “The Peroxisome: An Update on Mysteries 3.0,” Histochemistry and Cell Biology 161 (2024): 99–132, 10.1007/S00418-023-02259-5. 38244103 PMC10822820

[29] Y. Fujiki , Y. Abe , Y. Imoto , et al., “Recent Insights Into Peroxisome Biogenesis and Associated Diseases,” Journal of Cell Science 133 (2020): jcs236943, 10.1242/JCS.236943/224757. 32393673

[30] A. Van Der Zand , J. Gent , I. Braakman , and H. F. Tabak , “Biochemically Distinct Vesicles From the Endoplasmic Reticulum Fuse to Form Peroxisomes,” Cell 149 (2012): 397–409, 10.1016/j.cell.2012.01.054. 22500805

[31] C. Deisenroth , A. R. Thorner , T. Enomoto , C. M. Perou , and Y. Zhang , “Mitochondrial HEP27 Is a c‐Myb Target Gene That Inhibits Mdm2 and Stabilizes p53,” Molecular and Cellular Biology 30 (2010): 3981–3993, 10.1128/mcb.01284-09. 20547751 PMC2916441

[32] Z. Li , Y. Tan , X. Li , et al., “DHRS2 Inhibits Cell Growth and Metastasis in Ovarian Cancer by Downregulation of CHKα to Disrupt Choline Metabolism,” Cell Death & Disease 13 (2022): 845, 10.1038/s41419-022-05291-w. 36192391 PMC9530226

[33] Z. Li , H. Liu , A. Bode , and X. Luo , “Emerging Roles of Dehydrogenase/Reductase Member 2 (DHRS2) in the Pathology of Disease,” European Journal of Pharmacology 898 (2021): 173972, 10.1016/j.ejphar.2021.173972. 33652058

[34] M. Poźniak , N. Porębska , M. A. Krzyścik , et al., “The Cytotoxic Conjugate of Highly Internalizing Tetravalent Antibody for Targeting FGFR1‐Overproducing Cancer Cells,” Molecular Medicine 27 (2021): 46, 10.1186/S10020-021-00306-2. 33962559 PMC8103757

[35] M. Pozniak , A. Sokolowska‐Wedzina , K. Jastrzebski , et al., “FGFR1 Clustering With Engineered Tetravalent Antibody Improves the Efficiency and Modifies the Mechanism of Receptor Internalization,” Molecular Oncology 14 (2020): 1998–2021, 10.1002/1878-0261.12740. 32511887 PMC7463352

[36] A. Sokolowska‐Wedzina , A. Borek , J. Chudzian , P. Jakimowicz , M. Zakrzewska , and J. Otlewski , “Efficient Production and Purification of Extracellular Domain of Human FGFR‐Fc Fusion Proteins From Chinese Hamster Ovary Cells,” Protein Expression and Purification 99 (2014): 50–57, 10.1016/j.pep.2014.03.012. 24727156

[37] Y. Yosaatmadja , A. V. Patterson , J. B. Smaill , and C. J. Squire , “The 1.65 Å Resolution Structure of the Complex of AZD4547 With the Kinase Domain of FGFR1 Displays Exquisite Molecular Recognition,” Acta Crystallographica. Section D, Biological Crystallography 71 (2015): 525–533, 10.1107/S1399004714027539. 25760602

[38] D. Zukowska , A. Gedaj , N. Porebska , et al., “Receptor Clustering by a Precise Set of Extracellular Galectins Initiates FGFR Signaling,” Cellular and Molecular Life Sciences 80 (2023): 113, 10.1007/S00018-023-04768-X. 37012400 PMC10070233

[39] F. Gabrielli and S. Tofanelli , “Molecular and Functional Evolution of Human DHRS2 and DHRS4 Duplicated Genes,” Gene 511 (2012): 461–469, 10.1016/j.gene.2012.09.013. 23036705

[40] S. Pellegrini , S. Censini , S. Guidotti , et al., “A Human Short‐Chain Dehydrogenase/Reductase Gene: Structure, Chromosomal Localization, Tissue Expression and Subcellular Localization of Its Product,” Biochimica et Biophysica Acta—Gene Structure and Expression 1574 (2002): 215–222, 10.1016/S0167-4781(01)00323-2. 11997086

[41] N. Pfanner , B. Warscheid , and N. Wiedemann , “Mitochondrial Proteins: From Biogenesis to Functional Networks,” Nature Reviews. Molecular Cell Biology 20 (2019): 267–284, 10.1038/s41580-018-0092-0. 30626975 PMC6684368

[42] M. G. Claros and P. Vincens , “Computational Method to Predict Mitochondrially Imported Proteins and Their Targeting Sequences,” European Journal of Biochemistry 241 (1996): 779–786, 10.1111/j.1432-1033.1996.00779.x. 8944766

[43] J. J. A. Armenteros , M. Salvatore , O. Emanuelsson , et al., “Detecting Sequence Signals in Targeting Peptides Using Deep Learning,” Life Science Alliance 2 (2019): e201900429, 10.26508/lsa.201900429. 31570514 PMC6769257

[44] G. Neuberger , S. Maurer‐Stroh , B. Eisenhaber , A. Hartig , and F. Eisenhaber , “Prediction of Peroxisomal Targeting Signal 1 Containing Proteins From Amino Acid Sequence,” Journal of Molecular Biology 328 (2003): 581–592, 10.1016/S0022-2836(03)00319-X. 12706718

[45] J. Szymczyk , M. Sochacka , M. Biadun , K. D. Sluzalska , D. Witkowska , and M. Zakrzewska , “Overcoming Drug Resistance of Cancer Cells by Targeting the FGF1/FGFR1 Axis With Honokiol or FGF Ligand Trap,” Frontiers in Pharmacology 15 (2024): 1459820, 10.3389/FPHAR.2024.1459820/PDF. 39329123 PMC11424896

[46] M. Sochacka , L. Opalinski , J. Szymczyk , et al., “FHF1 Is a Bona Fide Fibroblast Growth Factor That Activates Cellular Signaling in FGFR‐Dependent Manner,” Cell Communication and Signaling 18 (2020): 69, 10.1186/S12964-020-00573-2. 32357892 PMC7193404

[47] A. Van Der Zand , I. Braakman , and H. F. Tabak , “Peroxisomal Membrane Proteins Insert Into the Endoplasmic Reticulum,” Molecular Biology of the Cell 21 (2010): 2057–2065, 10.1091/MBC.E10-02-0082. 20427571 PMC2883949

[48] H. F. Tabak , I. Braakman , and A. Van Der Zand , “Peroxisome Formation and Maintenance Are Dependent on the Endoplasmic Reticulum,” Annual Review of Biochemistry 82 (2013): 723–744, 10.1146/annurev-biochem-081111-125123. 23414306

[49] A. Van der Zand and H. F. Tabak , “Peroxisomes: Offshoots of the ER,” Current Opinion in Cell Biology 25 (2013): 449–454, 10.1016/j.ceb.2013.05.004. 23773570

[50] M. Schrader and L. Pellegrini , “The Making of a Mammalian Peroxisome, Version 2.0: Mitochondria Get Into the Mix,” Cell Death and Differentiation 24 (2017): 1148–1152, 10.1038/cdd.2017.23. 28409773 PMC5520164

[51] A. Akşit and I. J. van der Klei , “Yeast Peroxisomes: How Are They Formed and How Do They Grow?,” International Journal of Biochemistry and Cell Biology 105 (2018): 24–34, 10.1016/j.biocel.2018.09.019. 30268746

[52] M. Rudowitz and R. Erdmann , “Import and Quality Control of Peroxisomal Proteins,” Journal of Cell Science 136 (2023): jcs260999, 10.1242/JCS.260999. 37552037

[53] M. Katoh , Y. Loriot , G. Brandi , S. Tavolari , Z. A. Wainberg , and M. Katoh , “FGFR‐Targeted Therapeutics: Clinical Activity, Mechanisms of Resistance and New Directions,” Nature Reviews. Clinical Oncology 21 (2024): 312–329, 10.1038/s41571-024-00869-z. 38424198

[54] X. Yang , L. Ling , C. Li , et al., “STAMBPL1 Promotes the Progression of Lung Adenocarcinoma by Inhibiting DHRS2 Expression,” Translational Oncology 35 (2023): 101728, 10.1016/j.tranon.2023.101728. 37393834 PMC10331841

[55] X. Wu , Z. Zeng , K. Peng , D. Ren , and L. Zhang , “Regulatory Mechanism of DHRS2‐Modified Human Umbilical Cord Mesenchymal Stem Cells‐Derived Exosomes in Prostate Cancer Cell Proliferation and Apoptosis,” Tissue & Cell 82 (2023): 102078, 10.1016/j.tice.2023.102078. 37060745

[56] Y. Han , Z. Wang , S. Sun , et al., “Decreased DHRS2 Expression Is Associated With HDACi Resistance and Poor Prognosis in Ovarian Cancer,” Epigenetics 15 (2020): 122–133, 10.1080/15592294.2019.1656155. 31423895 PMC6961673

[57] X. Luo , N. Li , X. Zhao , et al., “DHRS2 Mediates Cell Growth Inhibition Induced by Trichothecin in Nasopharyngeal Carcinoma,” Journal of Experimental & Clinical Cancer Research 38 (2019): 300, 10.1186/s13046-019-1301-1. 31291971 PMC6617617

[58] Y. Zhou , L. Wang , X. Ban , et al., “DHRS2 Inhibits Cell Growth and Motility in Esophageal Squamous Cell Carcinoma,” Oncogene 37 (2018): 1086–1094, 10.1038/onc.2017.383. 29106393 PMC5851108

[59] Y. Zhao , C. Zhang , C. Zhang , et al., “SIRT3 Suppresses Vascular Endothelial Senescence via DHRS2 and Contributes to the Anti‐Vascular Aging Effect of Bazi Bushen Capsule,” Phytomedicine 140 (2025): 156571, 10.1016/j.phymed.2025.156571. 40049100

[60] A. Baker and R. Paudyal , “The Life of the Peroxisome: From Birth to Death,” Current Opinion in Plant Biology 22 (2014): 39–47, 10.1016/j.pbi.2014.09.003. 25261594

[61] M. R. Müller , A. Burmeister , M. A. Skowron , et al., “Characterization of the Dehydrogenase‐Reductase DHRS2 and Its Involvement in Histone Deacetylase Inhibition in Urological Malignancies,” Experimental Cell Research 439 (2024): 114055, 10.1016/j.yexcr.2024.114055. 38704080

