# Supplementary material for: Blockade of FGFR1 Trafficking to the Cell Surface Results in the Partial Mistargeting of the Receptor to Peroxisomes
Source: FASEB J. 2026 Jul 1;40(13):e72042. doi: 10.1096/fj.202600529RR (PMC13322175; doi:10.1096/fj.202600529RR)
Supplement: Supplementary file 1 — Figure S1: Panoramic view of the imaging of the immunofluorescence‐based co‐localization of wild‐type DHRS2 with the mitochondrial marker hsp60 and the peroxisomal marker protein PEX14 in U2OS‐SBP‐R1.GF cells. Scale bar represents 50 μm. Figure S2: Western blot confirmation of U2OS cell line transfection with DHRS2‐mGFP‐myc and myc‐mGFP‐DHRS2 using myc antibody. The asterisk indicates the endogenous myc protein. Figure S3: SDS‐PAGE analysis of purification of recombinant DHRS2 with C‐terminal StrepTagII from E. coli . DHRS2‐StrepTagII was purified using affinity chromatography. (A) Fractions collected during purification were resolved on SDS‐PAGE gel and visualized by Coomassie staining. Lane 1, molecular weight marker (kDa); Lane 2, cell pellet without induction (−IPTG); Lane 3, cell pellet with induction (+IPTG); Lane 4, insoluble fraction; Lane 5, soluble fraction; Lane 6, flow‐through; Lane 7, eluted fraction; Lane 8, fraction of the eluted protein after buffer exchange. The asterisk marks the target protein. (B) Confirmation of identity of DHRS2.StrepTagII using western blotting. Putative oligomeric forms of DHRS2.StrepTagII are marked with asterisks. Figure S4: (A) BLI analysis of the interaction between DHRS2 and kinase domain of FGFR1 (FGFR1‐KD). DHRS2 was immobilized on SAX biosensors and incubated with equal mass concentrations of recombinant FGFR1‐KD to record the association and dissociation phases. Empty sensor control values were subtracted from the signal obtained for FGFRs. Representative results from at least three independent experiments are shown. (B) BLI analysis of the interaction between DHRS2 and extracellular domain of FGFR1 fused to Fc antibody fragment (FGFR1‐Fc). DHRS2 was immobilized on SAX biosensors and incubated with equal mass concentrations of recombinant FGFR1‐Fc to record the association and dissociation phases. Empty sensor control values were subtracted from the signal obtained for FGFR1. Representative results from at least thre [file FSB2-40-e72042-s001.docx]

**Supplementary Information for:**

**Blockade of FGFR1 trafficking to the cell surface results in the partial mistargeting of the receptor to peroxisomes**

Paulina Działek^1#^, Aleksandra Chorążewska^1#^, Martyna Biaduń^2^, Jian Qiu^3^, Natalia Porębska^1^ and Łukasz Opaliński^1^*

^1^Department of Medical Biotechnology, Faculty of Biotechnology, University of Wroclaw, Joliot-Curie 14a, 50-383 Wroclaw, Poland

^2^Department of Protein Engineering, Faculty of Biotechnology, University of Wroclaw, Joliot-Curie 14a, 50-383 Wroclaw, Poland

^2^ Institute of Molecular Precision Medicine, Xiangya Hospital, Central South University, 410008 Changsha, China

^#^ These authors contributed equally to this work

*Correspondence should be addressed to Ł.O (lukasz.opalinski@uwr.edu.pl)

**Supplementary Figure Legends:**

**Figure S1.** Panoramic view of the imaging of the immunofluorescence-based co-localization of wild type DHRS2 with the mitochondrial marker hsp60 and the peroxisomal marker protein PEX14 in U2OS-SBP-R1.GF cells. Scale bar represents 50 μm.

**Figure S2.** Western blot confirmation of U2OS cell line transfection with DHRS2-mGFP-myc and myc-mGFP-DHRS2 using myc antibody. The asterisk indicates the endogenous myc protein.

**Figure S3.** SDS-PAGE analysis of purification of recombinant DHRS2 with C-terminal StrepTagII from *E. coli*. DHRS2-StrepTagII was purified using affinity chromatography. **A**. Fractions collected during purification were resolved on SDS-PAGE gel and visualized by Coomassie staining. Lane 1, Molecular weight marker (kDa); Lane 2, Cell pellet without induction (-IPTG); Lane 3, Cell pellet with induction (+IPTG); Lane 4, Insoluble fraction; Lane 5, Soluble fraction; Lane 6, Flow-through; Lane 7, Eluted fraction; Lane 8, Fraction of the eluted protein after buffer exchange. The asterisk marks the target protein. **B**. Confirmation of identity of DHRS2.StrepTagII using western blotting. Putative oligomeric forms of DHRS2.StrepTagII are marked with asterisks.

**Figure S4. A.** BLI analysis of the interaction between DHRS2 and kinase domain of FGFR1 (FGFR1-KD). DHRS2 was immobilized on SAX biosensors and incubated with equal mass concentrations of recombinant FGFR1-KD to record the association and dissociation phases. Empty sensor control values were subtracted from the signal obtained for FGFRs. Representative results from at least three independent experiments are shown. **B**. BLI analysis of the interaction between DHRS2 and extracellular domain of FGFR1 fused to Fc antibody fragment (FGFR1-Fc). DHRS2 was immobilized on SAX biosensors and incubated with equal mass concentrations of recombinant FGFR1-Fc to record the association and dissociation phases. Empty sensor control values were subtracted from the signal obtained for FGFR1. Representative results from at least three independent experiments are shown. **C.** Western blot confirmation of FGFR1-Fc deglycosylation after treatment with PNGase F. FGFR1-Fc was detected using anti-Fc antibody. The arrows indicate the mass shift. **D.** BLI analysis of the interaction between DHRS2 and PNGase F-deglycosylated FGFR1-Fc. PNGase F-deglycosylated FGFR1-Fc was immobilized on Protein-A biosensors and incubated with equal mass concentrations of DHRS2 to record the association and dissociation phases. Empty sensor control values were subtracted from the signal obtained for FGFR1.

**Figure S5**. Co-localization of SBP-FGFR1.GF with calnexin (ER marker) in U2OS-SBP-R1.GF cells and its analysis using quantitative confocal microscopy. Scale bar represents 10 μm or 2 μm for the zoomed fractions of the photos, respectively. Single dot represents co-localization percentage calculated in individual cell. At least 800 cells were analysed.

**Figure S6**. Co-localization of SBP-FGFR1.GF with PEX14 in U2OS-SBP-R1.GF cells. Scale bar represents 10 μm or 2 μm for the zoomed fractions of the photos, respectively.

**Table S1.** Summary of antibodies and fluorescent reagents used in this study. IF, immunofluorescence; WB, western blotting; PLA, proximity ligation assay.


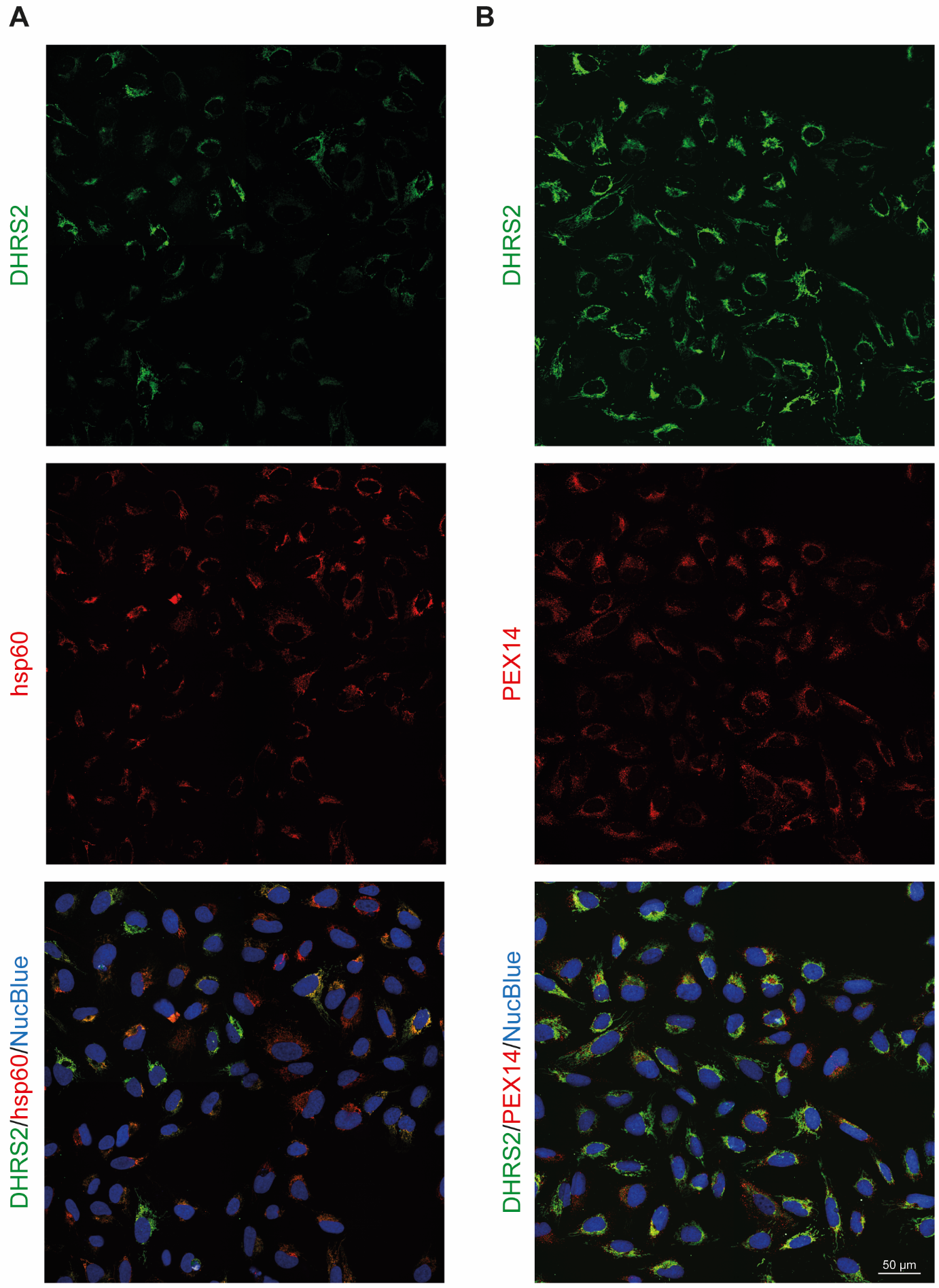


**Figure S1**

­­­­
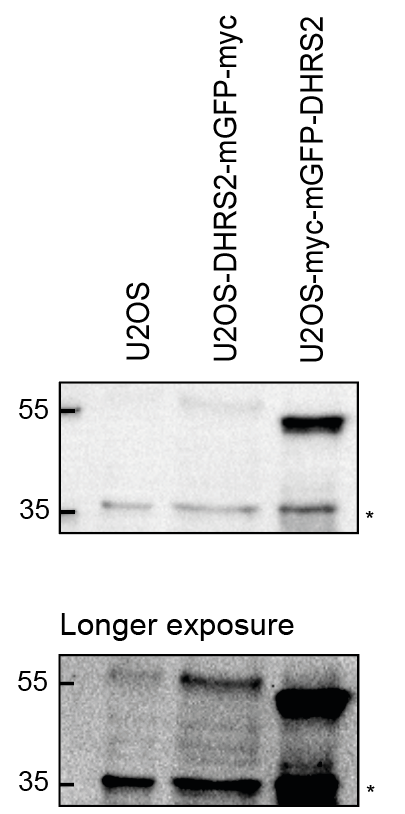


**Figure S2**

**
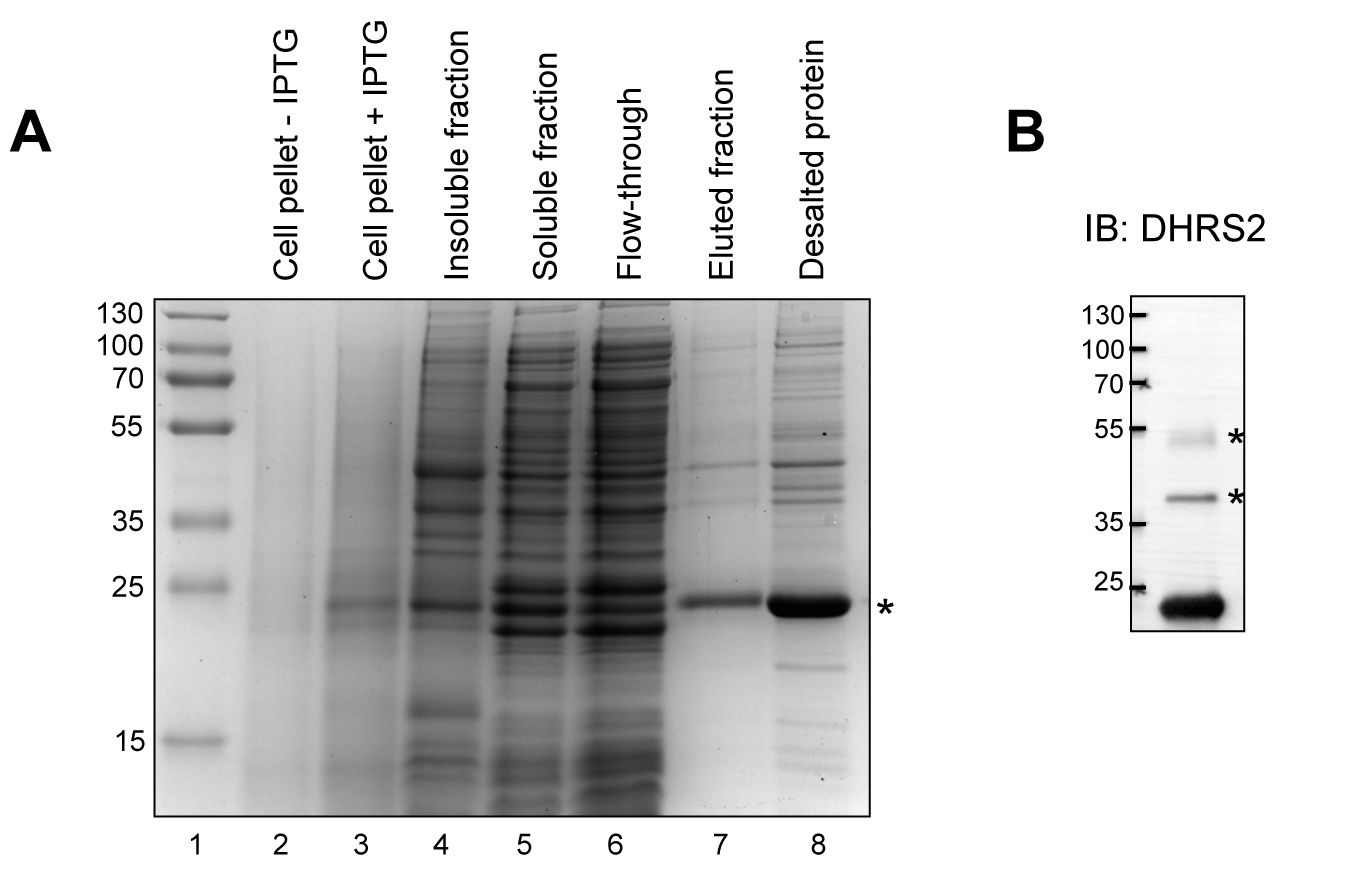
**

**Figure S3**

**
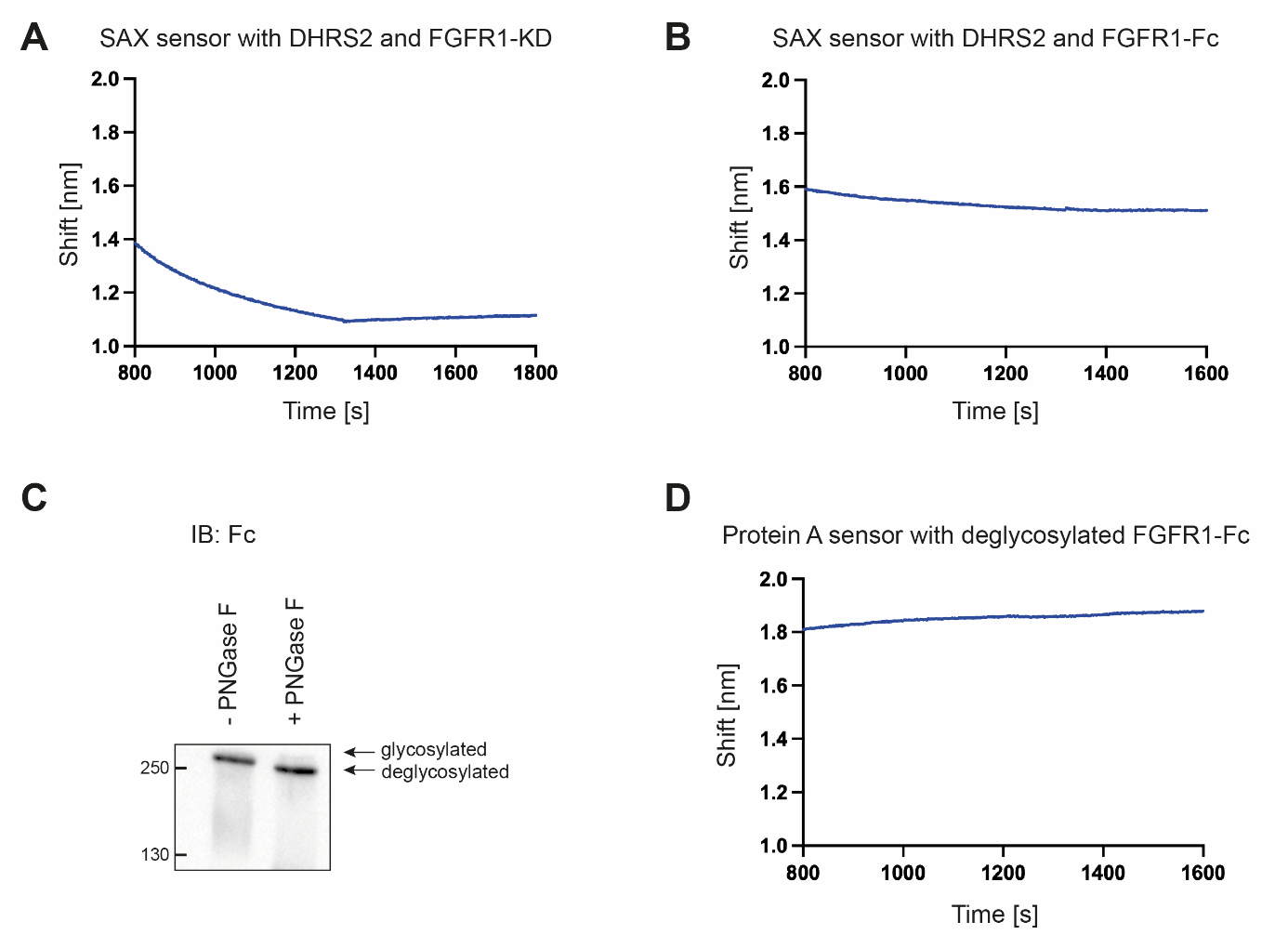
**

**Figure S4**

**
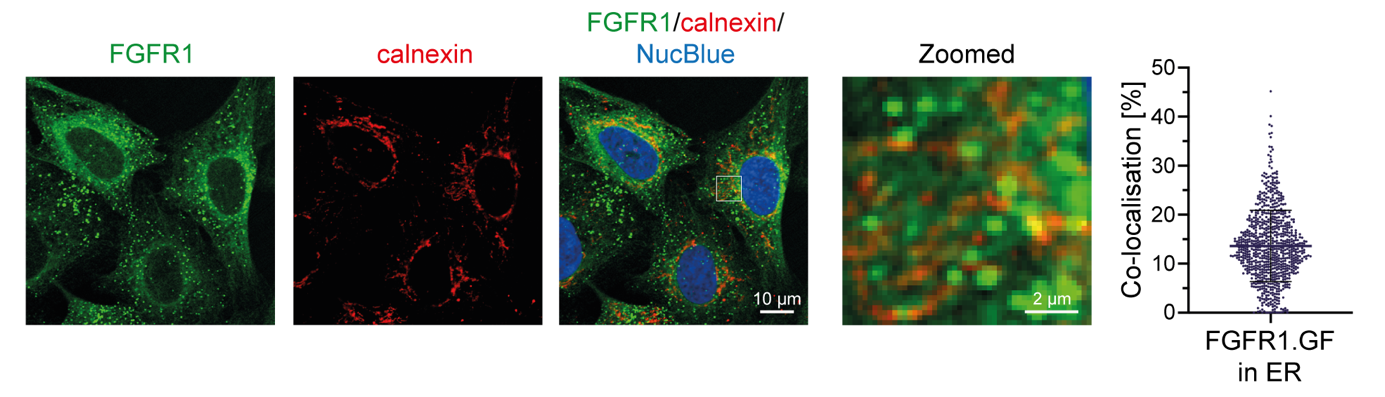
**

**Figure S5**

**
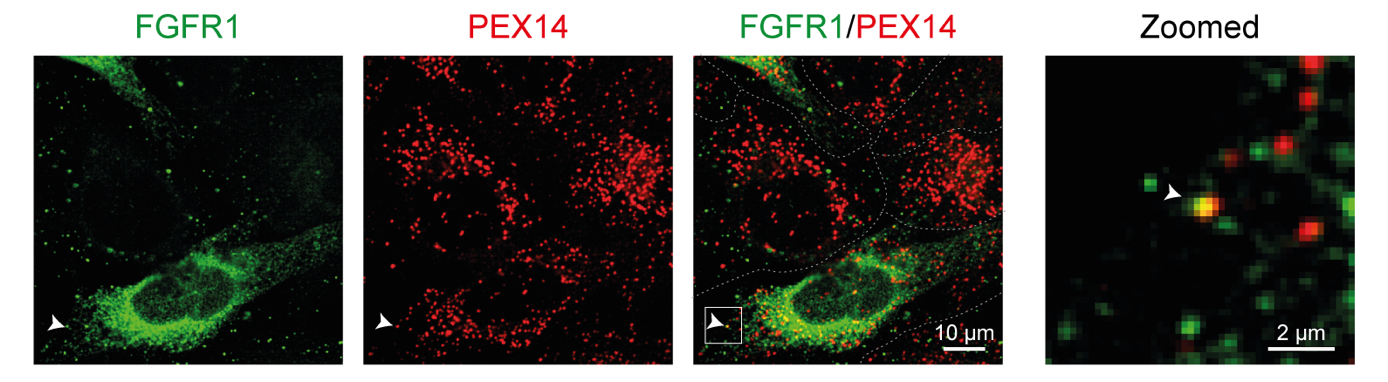
**

**Figure S6**

|  | **Antibody or reagent** | **Catalog #** | **Manufacturer** | **Application** |
| --- | --- | --- | --- | --- |
| **Primary antibodies** | Anti-FGFR1 antibody | 9740 | Cell Signaling | WB |
|  | Anti-phospho-FGFR antibody | 3476 | Cell Signaling | WB |
|  | Anti-ERK1/2 antibody | 9102 | Cell Signaling | WB |
|  | Anti-phospho-ERK1/2 antibody | 9101 | Cell Signaling | WB |
|  | Anti-catalase antibody | 12980 | Cell Signaling | IF |
|  | Anti-DHRS2 anttibody | 15735-1-AP | Proteintech | WB, IF |
|  | Anti-PEX14 antibody | 10594-1-AP | Proteintech | IF |
|  | Anti-cytochrome c antibody | sc-13156 | Santa Cruz Biotechnology | IF |
|  | Anti-Hsp60 antibody | sc-376261 | Santa Cruz Biotechnology | IF |
|  | Anti-SBP-tag antibody | sc-101595 | Santa Cruz Biotechnology | IF |
|  | Anti-PEX11b antibody | ab181066 | Abcam | WB, IF |
|  | Anti-calnexin antibody | #MA3-027 | Thermo Fisher Scientific | IF |
| **Secondary antibodies** | Anti-mouse HRP-conjugated antibody | 115-035-003 | Jackson Immuno-Research Laboratories | WB |
|  | Anti-rabbit HRP-conjugated antibody | 111-035-144 | Jackson Immuno-Research Laboratories | WB |
|  | Anti-rabbit antibody conjugated to Alexa Fluor 488 | 711-545-152 | Jackson Immuno-Research Laboratories | IF |
|  | Anti-rabbit antibody conjugated to Alexa Fluor 594 | A11037 | Thermo Fisher Scientific | IF |
|  | Anti-rabbit antibody conjugated to DyLight™ 650 | 84546 | Thermo Fisher Scientific | IF |
|  | Anti-mouse antibody conjugated to Alexa Fluor 488 | ab150077 | Abcam | IF |
|  | Anti-mouse antibody conjugated to Alexa Fluor 594 | 715-585-150 | Jackson Immuno-Research Laboratories | IF |
| **Reagents** | Zenon™ Human IgG Labeling Kit | Z25402 | Thermo Fisher Scientific | IF |
|  | CellLight™ Mitochondria-RFP, BacMam 2.0 | C10601 | Thermo Fisher Scientific | IF |
|  | HCS CellMask™ Deep Red Stain | H32721 | Thermo Fisher Scientific | IF |
|  | NucBlue™ Live ReadyProbes™ Reagent (Hoechst 33342) | R37605 | Thermo Fisher Scientific | IF |
|  | Duolink^®^ In Situ PLA^®^ Probe Anti-Mouse MINUS | DUO92004 | Sigma-Aldrich | IF, PLA |
|  | Duolink^®^ In Situ PLA^®^ Probe Anti-Rabbit PLUS | DUO92002 | Sigma-Aldrich | IF, PLA |
|  | Duolink^®^ In Situ Detection Reagents Red | DUO92008 | Sigma-Aldrich | IF, PLA |

**Table S1**
